# Covariance Representations and Coherent Measures for Some Entropies

**DOI:** 10.3390/e25111525

**Published:** 2023-11-07

**Authors:** Baishuai Zuo, Chuancun Yin

**Affiliations:** School of Statistics and Data Science, Qufu Normal University, Qufu 273165, China; bszuo124@163.com

**Keywords:** entropy, variability measure, covariance representation, coherent measure, shortfall, 91G70

## Abstract

We obtain covariance and Choquet integral representations for some entropies and give upper bounds of those entropies. The coherent properties of those entropies are discussed. Furthermore, we propose tail-based cumulative residual Tsallis entropy of order α (TCRTE) and tail-based right-tail deviation (TRTD); then, we define a shortfall of cumulative residual Tsallis (CRTES) and shortfall of right-tail deviation entropy (RTDS) and provide some equivalent results. As illustrated examples, the CRTESs of elliptical, inverse Gaussian, gamma and beta distributions are simulated.

## 1. Introduction

A risk measure is a functional ρ that maps from convex cone X of risks to $\bar{R}\equiv \left[-\infty ,+\infty \right]$ on a probability space (Ω,F,P). A good risk measure should satisfy some desirable properties (see, e.g., [1,2,3]). The several standard properties for general risk measures are presented as follows:

(A) Law invariance: For V,W∈X, if $V\overset{d}{=}W$, then ρ(V)=ρ(W);
(A1) Monotonicity: For V,W∈X, if V≤W, then ρ(V)≤ρ(W);
(A2) Translation invariance: For V∈X and any c∈R, we have ρ(V+c)=ρ(V)+c;
(A3) Positive homogeneity: For V∈X and any $\lambda \in R_{+}\equiv \left[0,\infty \right)$, we have ρ(λV)=λρ(V);
(A4) Subadditivity: For V,W∈X, we have ρ(V+W)≤ρ(V)+ρ(W);
(A5) Comonotonic additivity: If V and W are comonotonic, then ρ(V+W)=ρ(V)+ρ(W).

To estimate and identify risk measure, the law invariance (A) is an essential requirement. When a risk measure ρ further satisfies (A1)–(A4), then ρ is said to be coherent. It is well known that value-at-risk (VaR) and expected shortfall (ES) are the two extremely important risk measures used in banking and insurance. The VaR and ES at confidence level p∈(0,1) for a random variable (r.v.) *V* with cumulative distribution function (cdf) $F_{V}$ are defined as

$${\mathrm{VaR}}_{p}\left(V\right)=F_{V}^{-1}\left(p\right):=\inf\left\{p:F_{V}\left(x\right)\geq p\right\},$$

and

(1) $$\begin{array}{c} {\mathrm{ES}}_{p}\left(V\right)=\frac{1}{1-p}\int_{p}^{1}{\mathrm{VaR}}_{q}\left(V\right)dq, \end{array}$$

respectively. If $F_{V}$ is continuous, then ES equals the tail conditional expectation (TCE), which is written as

$$\begin{array}{c} {\mathrm{TCE}}_{p}\left(V\right)=E\left[V|V>v_{p}\right], \end{array}$$

where $v_{p}={\mathrm{VaR}}_{p}\left(V\right)$.

Some risk measures have other desirable properties; for example, (B1) Standardization: For c∈R, we have ν(c)=0; (B2) Location invariance: For V∈X and c∈R, we have ν(V+c)=ν(V). If a functional $\nu :V\to {\bar{R}}_{+}\equiv \left[0,\infty \right]$ satisfies law invariance (A), (B1) and (B2), we say that ν is a measure of variability. If ν further satisfies properties (A3) and (A4), then we say ν is a coherent measure of variability.

To capture the variability of the risk *V* beyond the quantile $v_{p}$, Furman and Landsman [4] proposed the tail standard deviation (TSD) risk measure

(2) $$\begin{array}{c} {\mathrm{TSD}}_{p}^{\lambda }\left(V\right)={\mathrm{TCE}}_{p}\left(V\right)+\lambda {\mathrm{SD}}_{p}\left(V\right), \end{array}$$

where p∈(0,1), λ≥0 denotes the loading parameter and ${\mathrm{SD}}_{p}\left(V\right)$ the tail standard deviation measure defined as

$$\begin{array}{c} {\mathrm{SD}}_{p}\left(V\right)=\sqrt{{\mathrm{TV}}_{p}\left(V\right)}. \end{array}$$

Here, ${\mathrm{TV}}_{p}\left(V\right)=E\left[{\left(V-{\mathrm{TCE}}_{p}\left(V\right)\right)}^{2}|V>v_{p}\right]$ is the tail variance of *V*. As its extension, Furman et al. [5] introduced the Gini shortfall (GS), which is defined by

(3) $$\begin{array}{c} {\mathrm{GS}}_{p}^{\lambda }\left(V\right)={\mathrm{ES}}_{p}\left(V\right)+\lambda {\mathrm{TGini}}_{p}\left(V\right), \end{array}$$

where ${\mathrm{TGini}}_{p}\left(V\right)=\frac{2}{{\left(1-p\right)}^{2}}\int_{p}^{1}F_{V}^{-1}\left(s\right)\left(2s-\left(1+p\right)\right)ds$ is tail-Gini functional. Recently, Hu and Chen [6] further proposed a shortfall of cumulative residual entropy (CRE), defined by

(4) $$\begin{array}{c} {\mathrm{CRES}}_{p}^{\lambda }\left(V\right)={\mathrm{ES}}_{p}\left(V\right)+\lambda {\mathrm{TCRE}}_{p}\left(V\right), \end{array}$$

where ${\mathrm{TCRE}}_{p}\left(V\right)=\int_{0}^{1}F_{V}^{-1}\left(s\right)dh_{v_{p}}\left(s\right)$ is the tail-based CRE of *V*. Here, $h_{v_{p}}\left(s\right)=0$ for s∈[0,p), and $h_{v_{p}}\left(s\right)=\left(\frac{1-s}{1-p}\right)\log\left(\frac{1-s}{1-p}\right)$ for s∈[p,1].

Inspired by those works, our main motivation is to find coherent shortfalls of entropy, which is the generalization of TSD, GS and CRES. These shortfalls of entropy can be used to capture the variability of a financial position. For specific financial applications, we can refer to [5,7,8]. To this aim, we give covariance and Choquet integral representations for some entropies, and provide upper bounds of those entropies. These representations not only make it easier for us to judge their cohesiveness, but also facilitate the extension of these results to two-dimensional and multi-dimensional cases in the future. Furthermore, we define TCRTE and TRTD, and propose CRTES and RTDS. As illustrated examples, CRTESs of elliptical, inverse Gaussian, gamma and beta distributions are simulated.

The remainder of this paper is structured as follows. Section 2 provides the covariance and Choquet integral representations for some entropies. Section 3 introduces some tail-based entropies. In Section 4, we propose two shortfalls of entropy, and give some equivalent results. The CRTESs of some parametric distributions are presented in Section 5. Finally, Section 6 concludes this paper and summarizes two possible research studies in the future.

Throughout the paper, let (Ω,F,P) be an atomless probability space. For a random variable *V* with cumulative distribution function (cdf) $F_{V}$, we use $U_{V}$ to denote any uniform [0,1] random variable such that $F_{V}^{-1}\left(U_{V}\right)=V$ holds almost everywhere. Let $L^{k}=L^{k}\left(\Omega ,F,P\right),k\in R_{+},$ be the set of all random variables on (Ω,F,P) with a finite *k*th-moment. Denote by $L_{+}^{0}$ the set of all non-negative random variables. $g^{\prime }$ denotes the first derivative of *g*. Notation $v_{+}=\max\left\{v,0\right\}$, and $1_{A}\left(\cdot \right)$ is the indicator function of set *A*.

## 2. Covariance and Choquet Integral Representations for Some Entropies

In this section, we derive covariance and Choquet integral representations for some entropies, which include initial, weighted and dynamic entropies. In addition, the upper bounds of these entropies are established.

Given *g* defined in [0,1] with g(0)=g(1)=0, weighted function ψ(·) and a r.v. *X* with cdf $F_{X}$, the initial and weighted entropies (forms) are defined as, respectively,

$$\begin{array}{c} \int_{-\infty }^{+\infty }g\left(F_{X}\left(x\right)\right)dx, \end{array}$$

and

$$\begin{array}{c} \int_{-\infty }^{+\infty }\psi \left(x\right)g\left(F_{X}\left(x\right)\right)dx. \end{array}$$

Further, given two r.v.s $X_{t}=\left[X-t|X>t\right]$ and $X_{\left(t\right)}=\left[X|X\leq t\right]$, the dynamic entropies (forms) are defined as

$$\begin{array}{c} \int_{-\infty }^{+\infty }g\left(F_{X_{t}}\left(x\right)\right)dx, \end{array}$$

and

$$\begin{array}{c} \int_{-\infty }^{+\infty }g\left(F_{X_{\left(t\right)}}\left(x\right)\right)dx. \end{array}$$

To derive the covariance of entropy, we first introduce below lemma.

**Lemma** **1** ([5]). *Let g be an almost everywhere (a.e.) differentiable function in [0,1] with $l=g^{\prime }$ (a.e.) and g(0)=g(1)=0. Suppose that $X\in L^{2}$ and $l\in L^{2}$. Then, we have*

$$\begin{array}{c} \int_{0}^{1}F_{X}^{-1}\left(v\right)dg\left(v\right)=\mathrm{Cov}\left(X,l\left(U_{X}\right)\right). \end{array}$$

*Further,*

$$\begin{array}{c} \int_{0}^{1}F_{X}^{-1}\left(v\right)dg\left(v\right)\leq \sqrt{\mathrm{Var}(X)}\sqrt{\mathrm{Var}(l\left(U_{X}\right))}. \end{array}$$

**Proof.**  Since *g* is almost everywhere differentiable in [0,1], we let $l\left(\cdot \right)=g^{\prime }\left(\cdot \right)\;\left(a.e.\right)$. Then,

$$\begin{array}{c} \int_{0}^{1}F_{X}^{-1}\left(v\right)dg\left(v\right)=\int_{0}^{1}F_{X}^{-1}\left(v\right)l\left(v\right)dv=E\left[Xl\left(U_{X}\right)\right]. \end{array}$$

Note that $E\left[l\left(U_{X}\right)\right]=\int_{0}^{1}l\left(v\right)dv=g\left(1\right)-g\left(0\right)=0$. Therefore,

$$\begin{array}{c} \int_{0}^{1}F_{X}^{-1}\left(v\right)dg\left(v\right)=\mathrm{Cov}\left(X,l\left(U_{X}\right)\right). \end{array}$$

Further, we use correlation coefficient $\varrho =\frac{\mathrm{Cov}(X,l\left(U_{X}\right))}{\sqrt{\mathrm{Var}(X)}\sqrt{\mathrm{Var}(l\left(U_{X}\right))}}$, −1≤ϱ≤1, and the last inequality is immediately obtained. □

### 2.1. Initial Entropy

To find the covariance represent of initial entropy, we give the following theorem.

**Theorem** **1.** *Let g be a continuous and almost everywhere differentiable function in [0,1] with $l=g^{\prime }$ (a.e.) and g(0)=g(1)=0. Further, there exists a unique minimum (or maximum) point $t_{0}\in \left(0,1\right)$ such that g is decreasing on $\left[0,t_{0}\right]$ and increasing on $\left[t_{0},1\right]$ (or there exists $t_{0}\in \left(0,1\right)$ such that g is increasing on $\left[0,t_{0}\right]$ and decreasing on $\left[t_{0},1\right]$). Suppose that $X\in L^{2}$ and $l\in L^{2}$. Then, we have*

(5) $$\begin{array}{cc} \int_{-\infty }^{+\infty }g\left(F_{X}\left(x\right)\right)dx & =-\int_{0}^{1}F_{X}^{-1}\left(u\right)dg\left(u\right)=-\mathrm{Cov}\left(X,l\left(U_{X}\right)\right). \end{array}$$

*Further,*

$$\begin{array}{c} \int_{-\infty }^{+\infty }g\left(F_{X}\left(x\right)\right)dx\leq \sqrt{\mathrm{Var}(X)}\sqrt{\mathrm{Var}(l\left(U_{X}\right))}. \end{array}$$

**Proof.**  Since g(u) is almost everywhere differentiable in [0,1], and g(0)=g(1)=0, there exists a unique minimum (or maximum) point $t_{0}\in \left(0,1\right)$. Hence, we can use *g* to induce Lebesgue–Stieltjes measures on the Borel-measurable spaces $\left(\left[0,t_{0}\right],B\left(\left[0,t_{0}\right]\right)\right)$ and $\left(\left[t_{0},1\right],B\left(\left[t_{0},1\right]\right)\right)$, respectively. Denote $x_{0}=F_{X}^{-1}\left(t_{0}\right)$; we have

$$\begin{array}{cc} \int_{-\infty }^{+\infty }g\left(F_{X}\left(x\right)\right)dx & =\int_{-\infty }^{x_{0}}g\left(F_{X}\left(x\right)\right)dx+\int_{x_{0}}^{+\infty }g\left(F_{X}\left(x\right)\right)dx\\ \; & =\int_{-\infty }^{x_{0}}\left(\int_{\left(0,F_{X}\left(x\right)\right]}dg\left(t\right)\right)dx-\int_{x_{0}}^{+\infty }\left(\int_{\left(F_{X}\left(x\right),1\right]}dg\left(t\right)\right)dx\\ \; & =-\int_{0}^{t_{0}}\left[F_{X}^{-1}\left(t\right)-x_{0}\right]dg\left(t\right)-\int_{t_{0}}^{1}\left[F_{X}^{-1}\left(t\right)-x_{0}\right]dg\left(t\right)\\ \; & =-\int_{0}^{1}F_{X}^{-1}\left(u\right)dg\left(u\right), \end{array}$$

where we have used Fubini’s theorem in the third equality. Further, using Lemma 1, we obtain

$$\begin{array}{c} -\int_{0}^{1}F_{X}^{-1}\left(u\right)dg\left(u\right)=-\mathrm{Cov}\left(X,l\left(U_{X}\right)\right). \end{array}$$

□

**Remark** **1.** *Note that the function g is of bounded variation since g has the following representation $g=g_{1}+g_{2}$, where $g_{1}$ is increasing and $g_{2}$ is decreasing. Similar results can be found in Lemma 3 of [9]. However, the result of this article is different from Lemma 3 of [9]. The integral interval and integrand are different, with one integrand being a function of F (i.e., g(F)) and the other being a function of $\bar{F}$ (i.e., $g(\bar{F})$). So, our result cannot be obtained from theirs.*

**Corollary** **1.** *Let g be a concave function in [0,1] with $l=g^{\prime }$ (a.e.) and g(0)=g(1)=0. Suppose that $X\in L^{2}$ and $l\in L^{2}$. Then, we have*

(6) $$\begin{array}{cc} \int_{-\infty }^{+\infty }g\left(F_{X}\left(x\right)\right)dx & =-\int_{0}^{1}F_{X}^{-1}\left(u\right)dg\left(u\right)=-\mathrm{Cov}\left(X,l\left(U_{X}\right)\right). \end{array}$$

*Hence,*

$$\begin{array}{c} \int_{-\infty }^{+\infty }g\left(F_{X}\left(x\right)\right)dx\leq \sqrt{\mathrm{Var}(X)}\sqrt{\mathrm{Var}(l\left(U_{X}\right))}. \end{array}$$

### 2.2. Weighted Entropy

Weighted entropy is an extension of initial entropy, which is an initial entropy associated with a weight function. We have the corresponding theorem as follows.

**Theorem** **2.** *Let g be a continuous and almost everywhere differentiable function in [0,1] with $l=g^{\prime }$ (a.e.) and g(0)=g(1)=0. Further, there exists a unique minimum (or maximum) point $t_{0}\in \left(0,1\right)$ and $\psi \left(t\right)=\Psi^{\prime }\left(t\right)$. Suppose that $\Psi \left(X\right)\in L^{2}$ and $l\in L^{2}$. Then, we have*

(7) $$\begin{array}{c} \int_{-\infty }^{+\infty }\psi \left(x\right)g\left(F_{X}\left(x\right)\right)dx=-\int_{0}^{1}\Psi \left(F_{X}^{-1}\left(u\right)\right)dg\left(u\right)=-\mathrm{Cov}\left[\Psi \left(X\right),l\left(U_{X}\right)\right]. \end{array}$$

*Further,*

$$\begin{array}{c} \int_{-\infty }^{+\infty }\psi \left(x\right)g\left(F_{X}\left(x\right)\right)dx\leq \sqrt{\mathrm{Var}(\Psi (X))}\sqrt{\mathrm{Var}(l\left(U_{X}\right))}. \end{array}$$

**Proof.**  Similar to the proof of Theorem 1, we have

$$\begin{array}{cc} \int_{-\infty }^{+\infty }\psi \left(x\right)g\left(F_{X}\left(x\right)\right)dx & =\int_{-\infty }^{x_{0}}\psi \left(x\right)g\left(F_{X}\left(x\right)\right)dx+\int_{x_{0}}^{+\infty }\psi \left(x\right)g\left(F_{X}\left(x\right)\right)dx\\ \; & =\int_{-\infty }^{x_{0}}\psi \left(x\right)\left(\int_{\left(0,F_{X}\left(x\right)\right]}dg\left(t\right)\right)dx-\int_{x_{0}}^{+\infty }\psi \left(x\right)\left(\int_{\left(F_{X}\left(x\right),1\right]}dg\left(t\right)\right)dx\\ \; & =\int_{0}^{t_{0}}\left(\int_{F_{X}^{-1}\left(t\right)}^{x_{0}}\psi \left(x\right)dx\right)dg\left(t\right)-\int_{t_{0}}^{1}\left(\int_{x_{0}}^{F_{X}^{-1}\left(t\right)}\psi \left(x\right)dx\right)dg\left(t\right)\\ \; & =-\int_{0}^{1}\Psi \left(F_{X}^{-1}\left(u\right)\right)dg\left(u\right), \end{array}$$

where we have used Fubini’s theorem in the third equality.

Note that

$$\begin{array}{cc} -\int_{0}^{1}\Psi \left(F_{X}^{-1}\left(u\right)\right)dg\left(u\right) & =-\int_{0}^{1}\Psi \left(F_{X}^{-1}\left(u\right)\right)l\left(u\right)du=-E\left[\Psi \left(X\right)l\left(U_{X}\right)\right], \end{array}$$

and $E\left[l\left(U_{X}\right)\right]=\int_{0}^{1}l\left(u\right)du=g\left(1\right)-g\left(0\right)=0$.

Therefore, we obtain

$$\begin{array}{c} -\int_{0}^{1}\Psi \left(F_{X}^{-1}\left(u\right)\right)dg\left(u\right)=-\mathrm{Cov}\left[\Psi \left(X\right),l\left(U_{X}\right)\right], \end{array}$$

ending the proof. □

**Corollary** **2.** *Let ψ(x)=x in Theorem 2; we have*

$$\begin{array}{cc} \int_{-\infty }^{+\infty }xg\left(F_{X}\left(x\right)\right)dx & =-\frac{1}{2}\int_{0}^{1}{\left[F_{X}^{-1}\left(u\right)\right]}^{2}dg\left(u\right)=-\frac{1}{2}\mathrm{Cov}\left(X^{2},l\left(U_{X}\right)\right). \end{array}$$

*Further,*

$$\begin{array}{c} \int_{-\infty }^{+\infty }xg\left(F_{X}\left(x\right)\right)dx\leq \frac{1}{2}\sqrt{\mathrm{Var}(X^{2})}\sqrt{\mathrm{Var}(l\left(U_{X}\right))}. \end{array}$$

**Corollary** **3.** *Let g be a concave function in [0,1] with $l=g^{\prime }$ (a.e.) and g(0)=g(1)=0. Suppose that $X^{2}\in L^{2}$ and $l\in L^{2}$. Then, we have*

(8) $$\begin{array}{cc} \int_{-\infty }^{+\infty }xg\left(F_{X}\left(x\right)\right)dx & =-\frac{1}{2}\int_{0}^{1}{\left[F_{X}^{-1}\left(u\right)\right]}^{2}dg\left(u\right)=-\frac{1}{2}\mathrm{Cov}\left(X^{2},l\left(U_{X}\right)\right). \end{array}$$

*Further,*

$$\begin{array}{c} \int_{-\infty }^{+\infty }xg\left(F_{X}\left(x\right)\right)dx\leq \frac{1}{2}\sqrt{\mathrm{Var}(X^{2})}\sqrt{\mathrm{Var}(l\left(U_{X}\right))}. \end{array}$$

### 2.3. Dynamic (Weighted) Entropy

Dynamic entropy is also a generalization of an initial entropy that focuses on feasible choices of the ranges (upper tail or lower tail).

The survival function of a random variable $X_{t}=\left[X-t|X>t\right]$ can be represented as

$$\begin{array}{c} {\bar{F}}_{X_{t}}\left(x\right)=\left\{\begin{array}{c} \frac{{\bar{F}}_{X}\left(x\right)}{{\bar{F}}_{X}\left(t\right)},\;\mathrm{when}\;x>t,\\ 1,\;\mathrm{otherwise}. \end{array}\right.\Leftrightarrow F_{X_{t}}\left(x\right)=\left\{\begin{array}{c} \frac{F_{X}\left(x\right)-F_{X}\left(t\right)}{1-F_{X}\left(t\right)},\;\mathrm{when}\;x>t,\\ 0,\;\mathrm{otherwise}. \end{array}\right. \end{array}$$

Therefore, for any v∈(0,1),

(9) $$\begin{array}{cc} F_{X_{t}}^{-1}\left(x\right) & =\inf\left\{x\in R:\frac{F_{X}\left(x\right)-F_{X}\left(t\right)}{1-F_{X}\left(t\right)}\geq v\right\}=\inf\left\{x\in R:F_{X}\left(x\right)\geq F_{X}\left(t\right)+\left(1-F_{X}\left(t\right)\right)v\right\}=F_{X}^{-1}\left(F_{X}\left(t\right)+\left(1-F_{X}\left(t\right)\right)v\right). \end{array}$$

**Theorem** **3.** *Let g be a continuous and almost everywhere differentiable function in [0,1] with $l=g^{\prime }$ (a.e.) and g(1)=g(0)=0. Further, there is a unique minimum (or maximum) point $t_{0}\in \left(0,1\right)$ and $\psi \left(t\right)=\Psi^{\prime }\left(t\right)$. Suppose that $\Psi \left(X\right)\in L^{2}$ and $l\in L^{2}$. Then, we have*

(10) $$\begin{array}{c} \int_{-\infty }^{+\infty }\psi \left(x\right)g\left(F_{X_{t}}\left(x\right)\right)dx=-\int_{0}^{1}\Psi \left(F_{X}^{-1}\left(v\right)\right)dg_{t}\left(v\right)=-\mathrm{Cov}\left[\Psi \left(X\right),l\left(\frac{F_{X}\left(X\right)-F_{X}\left(t\right)}{1-F_{X}\left(t\right)}\right)|X>t\right], \end{array}$$

*where $g_{t}\left(v\right)=0$ for $v\in [0,F_{X}\left(t\right))$, and $g_{t}\left(v\right)=h\left(\frac{v-F_{X}\left(t\right)}{1-F_{X}\left(t\right)}\right)$ for $v\in [F_{X}\left(t\right),1]$.*

**Proof.**  Using Equation (9) and the same argument of Theorem 2, we can easily obtain Theorem 3. □

**Corollary** **4.** *Let ψ(x)=1 in Theorem 3; we have*

(11) $$\begin{array}{cc} \int_{-\infty }^{+\infty }g\left(F_{X_{t}}\left(x\right)\right)dx\; & =-\int_{0}^{1}F_{X}^{-1}\left(v\right)dg_{t}\left(v\right)=-\mathrm{Cov}\left[X,l\left(\frac{F_{X}\left(X\right)-F_{X}\left(t\right)}{1-F_{X}\left(t\right)}\right)|X>t\right], \end{array}$$

*where $g_{t}\left(v\right)=0$ for $v\in [0,F_{X}\left(t\right))$, and $g_{t}\left(v\right)=g\left(\frac{v-F_{X}\left(t\right)}{1-F_{X}\left(t\right)}\right)$ for $v\in [F_{X}\left(t\right),1]$.*

**Corollary** **5.** *Let g be a concave function in [0,1] with $l=g^{\prime }$ (a.e.) and g(0)=g(1)=0. Suppose that $X\in L^{2}$ and $l\in L^{2}$. Then, we have*

(12) $$\begin{array}{cc} \int_{-\infty }^{+\infty }g\left(F_{X_{t}}\left(x\right)\right)dx\; & =-\int_{0}^{1}F_{X}^{-1}\left(u\right)dg_{t}\left(u\right)=-\mathrm{Cov}\left[X,l\left(\frac{F_{X}\left(X\right)-F_{X}\left(t\right)}{1-F_{X}\left(t\right)}\right)|X>t\right], \end{array}$$

*where $g_{t}\left(v\right)=0$ for $v\in [0,F_{X}\left(t\right))$, and $g_{t}\left(v\right)=g\left(\frac{v-F_{X}\left(t\right)}{1-F_{X}\left(t\right)}\right)$ for $v\in [F_{X}\left(t\right),1]$.*

The distribution function of a random variable $X_{\left(t\right)}=\left[X|X\leq t\right]$ can be written as

$$\begin{array}{c} F_{X_{\left(t\right)}}\left(x\right)=\left\{\begin{array}{c} \frac{F_{X}\left(x\right)}{F_{X}\left(t\right)},\;\mathrm{when}\;x\leq t,\\ 0,\;\mathrm{otherwise}. \end{array}\right. \end{array}$$

Therefore, for any s∈(0,1),

(13) $$\begin{array}{cc} F_{X_{\left(t\right)}}^{-1}\left(x\right) & =\inf\left\{x\in R:\frac{F_{X}\left(x\right)}{F_{X}\left(t\right)}\geq s\right\}=\inf\left\{x\in R:F_{X}\left(x\right)\geq F_{X}\left(t\right)s\right\}=F_{X}^{-1}\left(F_{X}\left(t\right)s\right). \end{array}$$

**Theorem** **4.** *Let g be a continuous and almost everywhere differentiable function in [0,1] with $l=g^{\prime }$ (a.e.) and g(1)=g(0)=0. Further, there is a unique minimum (or maximum) point $t_{0}\in \left(0,1\right)$ and $\psi \left(t\right)=\Psi^{\prime }\left(t\right)$. Suppose that $\Psi \left(X\right)\in L^{2}$ and $l\in L^{2}$. Then, we have*

(14) $$\begin{array}{cc} \int_{-\infty }^{+\infty }\psi \left(x\right)g\left(F_{X_{\left(t\right)}}\left(x\right)\right)dx\; & =-\int_{0}^{1}\Psi \left(F_{X}^{-1}\left(v\right)\right)dg_{\left(t\right)}\left(v\right)=-\mathrm{Cov}\left[\Psi \left(X\right),l\left(\frac{F_{X}\left(X\right)}{F_{X}\left(t\right)}\right)|X<t\right], \end{array}$$

*where $g_{\left(t\right)}\left(v\right)=0$ for $v\in (F_{X}\left(t\right),1]$, and $g_{\left(t\right)}\left(v\right)=g\left(\frac{v}{F_{X}\left(t\right)}\right)$ for $v\in [0,F_{X}\left(t\right)]$.*

**Proof.**  Using Equation (13), Theorem 2 and translation $v=F_{X}\left(t\right)u$, we can obtain Theorem 4. □

**Corollary** **6.** *Let ψ(x)=1 in Theorem 4; we have*

(15) $$\begin{array}{cc} \int_{-\infty }^{+\infty }g\left(F_{X_{\left(t\right)}}\left(x\right)\right)dx\; & =-\int_{0}^{1}F_{X}^{-1}\left(v\right)dg_{\left(t\right)}\left(v\right)=-\mathrm{Cov}\left[X,l\left(\frac{F_{X}\left(X\right)}{F_{X}\left(t\right)}\right)|X<t\right], \end{array}$$

*where $g_{\left(t\right)}\left(v\right)=0$ for $v\in (F_{X}\left(t\right),1]$, and $g_{\left(t\right)}\left(v\right)=g\left(\frac{v}{F_{X}\left(t\right)}\right)$ for $v\in [0,F_{X}\left(t\right)]$.*

**Corollary** **7.** *Let g be a concave function in [0,1] with $l=g^{\prime }$ (a.e.) and g(0)=g(1)=0. Suppose that $X\in L^{2}$ and $l\in L^{2}$. Then, we have*

(16) $$\begin{array}{cc} \int_{-\infty }^{+\infty }g\left(F_{X_{\left(t\right)}}\left(x\right)\right)dx\; & =-\int_{0}^{1}F_{X}^{-1}\left(u\right)dg_{\left(t\right)}\left(u\right)=-\mathrm{Cov}\left[X,l\left(\frac{F_{X}\left(X\right)}{F_{X}\left(t\right)}\right)|X<t\right], \end{array}$$

*where $g_{\left(t\right)}\left(v\right)=0$ for $v\in (F_{X}\left(t\right),1]$, and $g_{\left(t\right)}\left(v\right)=g\left(\frac{v}{F_{X}\left(t\right)}\right)$ for $v\in [0,F_{X}\left(t\right)]$.*

### 2.4. Examples

**Example** **1.** *Let $g\left(t\right)=\frac{1}{\alpha -1}\left(t-t^{\alpha }\right),\;\alpha >0,\;\alpha \neq 1,\;t\in \left[0,1\right]$, in Corollary 1, Equation (6) denoted by $CT_{\alpha }\left(X\right)$; we have*

$$\begin{array}{cc} CT_{\alpha }\left(X\right) & =\int_{-\infty }^{+\infty }\frac{1}{\alpha -1}\left(F_{X}\left(x\right)-{\left(F_{X}\left(x\right)\right)}^{\alpha }\right)dx=-\int_{0}^{1}F_{X}^{-1}\left(u\right)dg\left(u\right)=\frac{\alpha }{\alpha -1}\mathrm{Cov}\left(X,{\left(U_{X}\right)}^{\alpha -1}\right). \end{array}$$

*Further,*

$$\begin{array}{cc} CT_{\alpha }\left(X\right) & \leq |\frac{\alpha }{\alpha -1}|\sqrt{\mathrm{Var}(X)}\sqrt{\mathrm{Var}({\left(U_{X}\right)}^{\alpha -1})}=\frac{\sqrt{\mathrm{Var}(X)}}{\sqrt{2\alpha -1}},\;\alpha >\frac{1}{2}. \end{array}$$

In particular, when $X\in L_{+}^{0}$, the above measure denotes the cumulative Tsallis past entropy introduced by Calì et al. ([10]). Note that when α→1, it reduces to cumulative entropy (CE(X)), defined as (see [11])

(17) $$\begin{array}{cc} \mathrm{CE}(X)\; & =-\int_{0}^{+\infty }F_{X}\left(x\right)\log\left(F_{X}\left(x\right)\right)dx=\int_{0}^{1}F_{X}^{-1}\left(u\right)dh\left(u\right)=\mathrm{Cov}\left(X,\log\left(U_{X}\right)\right). \end{array}$$

Further,

$$\begin{array}{c} \mathrm{CE}\left(X\right)\leq \sqrt{\mathrm{Var}(X)}, \end{array}$$

where h(u)=ulogu.

In particular, when α=2, g(t)=t(1−t), we obtain

(18) $$\begin{array}{c} \mathrm{Gini}\left(X\right)=CT_{2}\left(X\right)=\int_{0}^{+\infty }F_{X}\left(x\right){\bar{F}}_{X}\left(x\right)dx=-\int_{0}^{1}F_{X}^{-1}\left(u\right)dg\left(u\right)=2\mathrm{Cov}\left(X,U_{X}\right). \end{array}$$

Further,

$$\begin{array}{c} \mathrm{Gini}\left(X\right)\leq \frac{\sqrt{\mathrm{Var}(X)}}{\sqrt{3}}, \end{array}$$

which is Gini mean semi-difference, denoted by Gini(X); for details, see [6,12].

**Example** **2.** *Let $g\left(t\right)=\frac{1}{\alpha -1}\left[\left(1-t\right)-{\left(1-t\right)}^{\alpha }\right],\;\alpha >0,\;\alpha \neq 1,\;t\in \left[0,1\right]$, in Corollary 1, Equation (6) denoted by $CRT_{\alpha }\left(X\right)$; we have*

$$\begin{array}{c} CRT_{\alpha }\left(X\right)=\int_{-\infty }^{+\infty }\frac{1}{\alpha -1}\left({\bar{F}}_{X}\left(x\right)-{\left({\bar{F}}_{X}\left(x\right)\right)}^{\alpha }\right)dx=-\int_{0}^{1}F_{X}^{-1}\left(u\right)dg\left(u\right)=-\frac{\alpha }{\alpha -1}\mathrm{Cov}\left(X,{\left(1-U_{X}\right)}^{\alpha -1}\right). \end{array}$$

*Further,*

$$\begin{array}{cc} CRT_{\alpha }\left(X\right) & \leq |\frac{\alpha }{\alpha -1}|\sqrt{\mathrm{Var}(X)}\sqrt{\mathrm{Var}({\left(1-U_{X}\right)}^{\alpha -1})}=\frac{\sqrt{\mathrm{Var}(X)}}{\sqrt{2\alpha -1}},\;\alpha >\frac{1}{2}. \end{array}$$

In particular, when $X\in L_{+}^{0}$, the above measure is the cumulative residual Tsallis entropy of order α introduced by Rajesh and Sunoj [13]. Note that when α→1, it reduces to cumulative entropy (E(X)), defined as (see [14])

(19) $$\begin{array}{c} E\left(X\right)=-\int_{0}^{+\infty }{\bar{F}}_{X}\left(x\right)\log\left({\bar{F}}_{X}\left(x\right)\right)dx=\int_{0}^{1}F_{X}^{-1}\left(u\right)dh\left(u\right)=-\mathrm{Cov}\left(X,\log\left(1-U_{X}\right)\right). \end{array}$$

Further,

$$\begin{array}{c} E\left(X\right)\leq \sqrt{\mathrm{Var}(X)}, \end{array}$$

where h(u)=(1−u)log(1−u).

**Example** **3.** *Let $g\left(t\right)=\left(1-t\right){\left[-\log\left(1-t\right)\right]}^{\alpha },\;0<\alpha \leq 1,\;t\in \left[0,1\right]$, in Corollary 1, Equation (6) denoted by $FE_{\alpha }\left(X\right)$, so that $l\left(t\right)={\left[-\log\left(1-t\right)\right]}^{\alpha -1}\left[\alpha +\log\left(1-t\right)\right]$. Then, we have*

$$\begin{array}{c} FE_{\alpha }\left(X\right)=\int_{-\infty }^{+\infty }{\bar{F}}_{X}\left(x\right){\left[-\log\left({\bar{F}}_{X}\left(x\right)\right)\right]}^{\alpha }dx=-\int_{0}^{1}F_{X}^{-1}\left(u\right)dg\left(u\right)=-\mathrm{Cov}\left(X,l\left(U_{X}\right)\right). \end{array}$$

*Further,*

$$\begin{array}{c} FE_{\alpha }\left(X\right)\leq \alpha \sqrt{\mathrm{Var}(X)}\sqrt{E[{\left(\log\left(1-U_{X}\right)\right)}^{2\alpha -2}]}. \end{array}$$

In particular, when X∈(0,c), the above measure is called the fractional cumulative residual entropy of *X* by Xiong et al. [15].

**Example** **4.** *Let $g\left(t\right)={\left(1-t\right)}^{1/2}-\left(1-t\right)$ in Corollary 1, Equation (6) denoted by D(X); we have*

$$\begin{array}{cc} D(X)\; & =\int_{-\infty }^{+\infty }\left[{\left({\bar{F}}_{X}\left(x\right)\right)}^{1/2}-{\bar{F}}_{X}\left(x\right)\right]dx=-\int_{0}^{1}F_{X}^{-1}\left(u\right)dg\left(u\right). \end{array}$$

In particular, when $X\in L_{+}^{0}$, the above measure is the right-tail deviation introduced by Wang [16].

**Example** **5.** *Let $g\left(t\right)=-2[t+{\left(1-t\right)}^{r}-1],\;r>1$, in Corollary 1, Equation (6) denoted by ${\mathrm{EGini}}_{r}\left(X\right)$; we have*

$$\begin{array}{cc} {\mathrm{EGini}}_{r}\left(X\right)=\int_{-\infty }^{+\infty }g\left(F_{X}\left(x\right)\right)dx & =-\int_{0}^{1}F_{X}^{-1}\left(u\right)dg\left(u\right)=-2r\mathrm{Cov}\left(X,{\left(1-U_{X}\right)}^{r-1}\right). \end{array}$$

*Further,*

$$\begin{array}{c} \int_{-\infty }^{+\infty }g\left(F_{X}\left(x\right)\right)dx\leq \frac{2(r-1)}{\sqrt{2r-1}}\sqrt{\mathrm{Var}(X)}. \end{array}$$

In particular, when $X\in L_{+}^{0}$, the above measure is the extended Gini coefficient (see [7]). As a special case, when r=2, the extended Gini coefficient becomes the simple Gini (see [5]).

**Example** **6.** *Let $g\left(t\right)=\frac{1}{\Gamma (\alpha +1)}\left(1-t\right){\left[-\log\left(1-t\right)\right]}^{\alpha },\;\alpha >0,\;t\in \left[0,1\right]$, in Theorem 1, Equation (5) denoted by $FGRE_{\alpha }\left(X\right)$, so that $l\left(t\right)=\frac{1}{\Gamma (\alpha +1)}{\left[-\log\left(1-t\right)\right]}^{\alpha -1}\left[\alpha +\log\left(1-t\right)\right]$. Then, we have*

$$\begin{array}{c} FGRE_{\alpha }\left(X\right)=\frac{1}{\Gamma (\alpha +1)}\int_{-\infty }^{+\infty }{\bar{F}}_{X}\left(x\right){\left[-\log\left({\bar{F}}_{X}\left(x\right)\right)\right]}^{\alpha }dx=-\int_{0}^{1}F_{X}^{-1}\left(u\right)dg\left(u\right)=-\mathrm{Cov}\left(X,l\left(U_{X}\right)\right). \end{array}$$

*Further,*

$$\begin{array}{c} FGE_{\alpha }\left(X\right)\leq \frac{1}{\Gamma (\alpha )}\sqrt{\mathrm{Var}(X)}\sqrt{E[{\left(\log\left(1-U_{X}\right)\right)}^{2\alpha -2}]}. \end{array}$$

In particular, when X∈(0,c), the above measure is called the fractional generalized cumulative residual entropy of *X* by Di Crescenzo et al. [17].

In particular, if α is a positive integer, say α=n∈N, in this case, $l\left(t\right)=\frac{1}{n!}{\left[-\log\left(1-t\right)\right]}^{n-1}\left[n+\log\left(1-t\right)\right]$. Then, $FGRE_{\alpha }\left(X\right)$ identifies with the generalized cumulative residual entropy ($GCRE_{n}\left(X\right)$) that has been introduced by Psarrakos and Navarro [18], i.e.,

$$\begin{array}{c} GCRE_{n}\left(X\right)=\frac{1}{n!}\int_{0}^{+\infty }{\bar{F}}_{X}\left(x\right){\left[-\log\left({\bar{F}}_{X}\left(x\right)\right)\right]}^{n}dx=-\int_{0}^{1}F_{X}^{-1}\left(u\right)dg\left(u\right)=-\mathrm{Cov}\left(X,l\left(U_{X}\right)\right). \end{array}$$

Further,

$$\begin{array}{c} GCRE_{n}\left(X\right)\leq \frac{\sqrt{(2n-2)!}}{(n-1)!}\sqrt{\mathrm{Var}(X)}. \end{array}$$

**Example** **7.** *Let $g\left(t\right)=\frac{1}{\Gamma (\alpha +1)}t{\left[-\log t\right]}^{\alpha },\;\alpha >0,\;t\in \left[0,1\right]$, in Theorem 1, Equation (5) denoted by $FGE_{\alpha }\left(X\right)$, so that $l\left(t\right)=\frac{1}{\Gamma (\alpha +1)}{\left[-\log\left(t\right)\right]}^{\alpha -1}\left[\alpha +\log\left(t\right)\right]$. Then, we have*

$$\begin{array}{c} FGE_{\alpha }\left(X\right)=\frac{1}{\Gamma (\alpha +1)}\int_{-\infty }^{+\infty }F_{X}\left(x\right){\left[-\log\left(F_{X}\left(x\right)\right)\right]}^{\alpha }dx=-\int_{0}^{1}F_{X}^{-1}\left(u\right)dg\left(u\right)=\mathrm{Cov}\left(X,l\left(U_{X}\right)\right). \end{array}$$

*Further,*

$$\begin{array}{c} FGE_{\alpha }\left(X\right)\leq \frac{1}{\Gamma (\alpha )}\sqrt{\mathrm{Var}(X)}\sqrt{E[{\left(\log\left(1-U_{X}\right)\right)}^{2\alpha -2}]}. \end{array}$$

In particular, when X∈(0,c), the above measure is called the fractional generalized cumulative entropy of *X* by Di Crescenzo et al. ([17]).

In particular, if α is a positive integer, say α=n∈N, in this case, $l\left(t\right)=\frac{1}{n!}{\left[-\log\left(t\right)\right]}^{n-1}$[n+log(t)]. Then, $FGE_{\alpha }\left(X\right)$ identifies with the generalized cumulative entropy ($GCE_{n}\left(X\right)$) that has been introduced by Kayal [19] (see also [20]), i.e.,

$$\begin{array}{cc} GCE_{n}\left(X\right) & =\frac{1}{n!}\int_{0}^{+\infty }F_{X}\left(x\right){\left[-\log\left(F_{X}\left(x\right)\right)\right]}^{n}dx=-\int_{0}^{1}F_{X}^{-1}\left(u\right)dg\left(u\right)=\mathrm{Cov}\left(X,l\left(U_{X}\right)\right). \end{array}$$

Further,

$$\begin{array}{c} GCE_{n}\left(X\right)\leq \frac{\sqrt{(2n-2)!}}{(n-1)!}\sqrt{\mathrm{Var}(X)}. \end{array}$$

**Example** **8.** *Let $g\left(t\right)=\frac{1}{\alpha -1}\left(t-t^{\alpha }\right),\;\alpha >0,\;\alpha \neq 1,\;t\in \left[0,1\right]$, in Corollary 3, Equation (8) denoted by $WCT_{\alpha }\left(X\right)$; we have*

$$\begin{array}{c} WCT_{\alpha }\left(X\right)=\int_{-\infty }^{+\infty }\frac{1}{\alpha -1}x\left[F_{X}\left(x\right)-{\left(F_{X}\left(x\right)\right)}^{\alpha }\right]dx=-\frac{1}{2}\int_{0}^{1}{\left[F_{X}^{-1}\left(u\right)\right]}^{2}dg\left(u\right)=\frac{\alpha }{2(\alpha -1)}\mathrm{Cov}\left(X^{2},{\left(U_{X}\right)}^{\alpha -1}\right). \end{array}$$

*Further,*

$$\begin{array}{cc} WCT_{\alpha }\left(X\right) & \leq |\frac{\alpha }{2(\alpha -1)}|\sqrt{\mathrm{Var}(X^{2})}\sqrt{\mathrm{Var}({\left(U_{X}\right)}^{\alpha -1})}=\frac{\sqrt{\mathrm{Var}(X^{2})}}{2\sqrt{2\alpha -1}},\;\alpha >\frac{1}{2}. \end{array}$$

In particular, when $X\in L_{+}^{0}$, the above measure is the weighted cumulative Tsallis entropy of order α introduced by Chakraborty and Pradhan [21]. Note that when α→1, it reduces to weighted cumulative entropy (${\mathrm{CE}}^{w}\left(X\right)$), defined as (see [22,23])

$$\begin{array}{cc} {\mathrm{CE}}^{w}\left(X\right) & =-\int_{0}^{+\infty }xF_{X}\left(x\right)\log\left(F_{X}\left(x\right)\right)dx=\frac{1}{2}\int_{0}^{1}{\left[F_{X}^{-1}\left(u\right)\right]}^{2}dh\left(u\right)=\frac{1}{2}\mathrm{Cov}\left(X^{2},\log\left(U_{X}\right)\right). \end{array}$$

Further,

$$\begin{array}{c} {\mathrm{CE}}^{w}\left(X\right)\leq \frac{1}{2}\sqrt{\mathrm{Var}(X^{2})}, \end{array}$$

where h(u)=ulogu.

In particular, when α=2, g(t)=t(1−t), we obtain

$$\begin{array}{c} WCT_{2}\left(X\right)=\int_{-\infty }^{+\infty }xF_{X}\left(x\right){\bar{F}}_{X}\left(x\right)dx=-\frac{1}{2}\int_{0}^{1}{\left[F_{X}^{-1}\left(u\right)\right]}^{2}dg\left(u\right)=\mathrm{Cov}\left(X^{2},U_{X}\right). \end{array}$$

Further,

$$\begin{array}{c} WCT_{2}\left(X\right)\leq \frac{\sqrt{\mathrm{Var}(X^{2})}}{2\sqrt{3}}. \end{array}$$

**Example** **9.** *Let $g\left(t\right)=\frac{1}{\alpha -1}\left[\left(1-t\right)-{\left(1-t\right)}^{\alpha }\right],\;\alpha >0,\;\alpha \neq 1,\;t\in \left[0,1\right]$, in Corollary 3, Equation (8) denoted by $WCRT_{\alpha }\left(X\right)$; we have*

$$\begin{array}{cc} WCRT_{\alpha }\left(X\right)\; & =\int_{-\infty }^{+\infty }\frac{1}{\alpha -1}x\left[{\bar{F}}_{X}\left(x\right)-{\left({\bar{F}}_{X}\left(x\right)\right)}^{\alpha }\right]dx=-\frac{1}{2}\int_{0}^{1}{\left[F_{X}^{-1}\left(u\right)\right]}^{2}dg\left(u\right)=-\frac{\alpha }{2(\alpha -1)}\mathrm{Cov}\left(X^{2},{\left(1-U_{X}\right)}^{\alpha -1}\right). \end{array}$$

*Further,*

$$\begin{array}{cc} WCRT_{\alpha }\left(X\right) & \leq |\frac{\alpha }{2(\alpha -1)}|\sqrt{\mathrm{Var}(X^{2})}\sqrt{\mathrm{Var}({\left(1-U_{X}\right)}^{\alpha -1})}=\frac{\sqrt{\mathrm{Var}(X^{2})}}{2\sqrt{2\alpha -1}},\;\alpha >\frac{1}{2}. \end{array}$$

In particular, when $X\in L_{+}^{0}$, the above measure is the weighted cumulative residual Tsallis entropy of order α introduced by Chakraborty and Pradhan [21]. Note that when α→1, it reduces to weighted cumulative residual entropy ($E^{w}\left(X\right)$), defined as (see [23,24])

$$\begin{array}{cc} E^{w}\left(X\right)\; & =-\int_{0}^{+\infty }x{\bar{F}}_{X}\left(x\right)\log\left({\bar{F}}_{X}\left(x\right)\right)dx=\frac{1}{2}\int_{0}^{1}{\left[F_{X}^{-1}\left(u\right)\right]}^{2}d\left(h\left(u\right)\right)=-\frac{1}{2}\mathrm{Cov}\left(X^{2},\log\left(1-U_{X}\right)\right). \end{array}$$

Further,

$$\begin{array}{c} E^{w}\left(X\right)\leq \frac{1}{2}\sqrt{\mathrm{Var}(X^{2})}, \end{array}$$

where h(u)=(1−u)log(1−u).

**Example** **10.** *Let $g\left(t\right)=\frac{1}{n!}\left(1-t\right){\left[-\log\left(1-t\right)\right]}^{n},\;t\in \left[0,1\right]$, in Theorem 2, Equation (7) denoted by $WGCRE_{n,\psi }\left(X\right)$, so that $l\left(t\right)=\frac{1}{n!}{\left[-\log\left(1-t\right)\right]}^{n-1}\left[n+\log\left(1-t\right)\right]$. Then, we have*

$$\begin{array}{c} WGCRE_{n,\psi }\left(X\right)=\frac{1}{n!}\int_{-\infty }^{+\infty }\psi \left(x\right){\bar{F}}_{X}\left(x\right){\left[-\log\left({\bar{F}}_{X}\left(x\right)\right)\right]}^{n}dx=-\int_{0}^{1}\Psi \left(F_{X}^{-1}\left(u\right)\right)dg\left(u\right)=-\mathrm{Cov}\left[\Psi \left(X\right),l\left(U_{X}\right)\right]. \end{array}$$

*Hence,*

$$\begin{array}{c} WGCRE_{n,\psi }\left(X\right)\leq \frac{\sqrt{(2n-2)!}}{(n-1)!}\sqrt{\mathrm{Var}(\Psi (X))}. \end{array}$$

In particular, when $X\in L_{+}^{0}$, the above measure is the weighted generalized cumulative residual entropy introduced by Tahmasebi ([25]) (also see [26]). As a special case, when ψ(x)=x, $WGCRE_{n,\psi }\left(X\right)$ reduces to a shift-dependent GCRE of order *n* ($WGCRE_{n}\left(X\right)$) defined by Kayal [27], i.e.,

$$\begin{array}{cc} WGCRE_{n}\left(X\right)\; & =\frac{1}{n!}\int_{0}^{+\infty }x{\bar{F}}_{X}\left(x\right){\left[-\log\left({\bar{F}}_{X}\left(x\right)\right)\right]}^{n}dx=-\frac{1}{2}\int_{0}^{1}{\left[F_{X}^{-1}\left(u\right)\right]}^{2}dg\left(u\right)=-\frac{1}{2}\mathrm{Cov}\left[X^{2},l\left(U_{X}\right)\right]. \end{array}$$

Further,

$$\begin{array}{c} WGCRE_{n}\left(X\right)\leq \frac{\sqrt{(2n-2)!}}{2[(n-1)!]}\sqrt{\mathrm{Var}(X^{2})}. \end{array}$$

In particular, when n=1, g(t)=(1−t)[−log(1−t)], the $WGCRE_{n,\psi }\left(X\right)$ reduces to weighted cumulative residual entropy with weight function ψ ($WGCRE_{\psi }\left(X\right)$) defined by Suhov and Yasaei Sekeh [28], i.e.,

$$\begin{array}{cc} WGCRE_{\psi }\left(X\right)\; & =\int_{0}^{+\infty }\psi \left(x\right){\bar{F}}_{X}\left(x\right)\left[-\log\left({\bar{F}}_{X}\left(x\right)\right)\right]dx=-\int_{0}^{1}\Psi \left(F_{X}^{-1}\left(u\right)\right)dg\left(u\right)=-\mathrm{Cov}\left[\Psi \left(X\right),\log\left(1-U_{X}\right)\right]. \end{array}$$

Further,

$$\begin{array}{c} WGCRE_{\psi }\left(X\right)\leq \sqrt{\mathrm{Var}(\Psi (X))}. \end{array}$$

They also define weighted cumulative entropy with weight function ψ ($WGCE_{\psi }\left(X\right)$; in this case, g(t)=t[−log(t)]):

$$\begin{array}{cc} WGCE_{\psi }\left(X\right)\; & =\int_{0}^{+\infty }\psi \left(x\right)F_{X}\left(x\right)\left[-\log\left(F_{X}\left(x\right)\right)\right]dx=-\int_{0}^{1}\Psi \left(F_{X}^{-1}\left(u\right)\right)dg\left(u\right)=\mathrm{Cov}\left[\Psi \left(X\right),\log\left(U_{X}\right)\right]. \end{array}$$

Further,

$$\begin{array}{c} WGCE_{\psi }\left(X\right)\leq \sqrt{\mathrm{Var}(\Psi (X))}. \end{array}$$

**Example** **11.** *Let g(u)=−(1−u)log(1−u) in Corollary 5, Equation (12) denoted by $DE_{t}\left(X\right)$; we have*

$$\begin{array}{cc} DE_{t}\left(X\right)\; & =-\int_{t}^{+\infty }\frac{{\bar{F}}_{X}\left(x\right)}{{\bar{F}}_{X}\left(t\right)}\log\left(\frac{{\bar{F}}_{X}\left(x\right)}{{\bar{F}}_{X}\left(t\right)}\right)dx=-\int_{0}^{1}F_{X}^{-1}\left(v\right)dg_{t}\left(v\right)=-\mathrm{Cov}\left[X,\log\left({\bar{F}}_{X}\left(X\right)\right)|X>t\right], \end{array}$$

*where $g_{t}\left(v\right)=0$ for $v\in [0,F_{X}\left(t\right))$, and $g_{t}\left(v\right)=-\left(\frac{1-v}{1-F_{X}\left(t\right)}\right)\log\left(\frac{1-v}{1-F_{X}\left(t\right)}\right)$ for $v\in [F_{X}\left(t\right),1]$.*

In particular, when $X\in L_{+}^{0}$, the above measure is dynamic cumulative entropy defined by Asadi and Zohrevand [29].

**Example** **12.** *Let $g\left(t\right)=\left(1-t\right){\left[-\log\left(1-t\right)\right]}^{\alpha },\;0<\alpha \leq 1,\;t\in \left[0,1\right]$ in Corollary 5, Equation (12) denoted by ${\mathrm{DFCRE}}_{\alpha ,t}\left(X\right)$, so that $l\left(t\right)={\left[-\log\left(1-t\right)\right]}^{\alpha -1}\left[\alpha +\log\left(1-t\right)\right]$. Then, we have*

$$\begin{array}{cc} {\mathrm{DFCRE}}_{\alpha ,t}\left(X\right)\; & =\int_{t}^{+\infty }\frac{{\bar{F}}_{X}\left(x\right)}{{\bar{F}}_{X}\left(t\right)}{\left[-\log\left(\frac{{\bar{F}}_{X}\left(x\right)}{{\bar{F}}_{X}\left(t\right)}\right)\right]}^{\alpha }dx=-\int_{0}^{1}F_{X}^{-1}\left(u\right)dg_{t}\left(u\right)=-\mathrm{Cov}\left[X,l\left(\frac{F_{X}\left(X\right)-F_{X}\left(t\right)}{1-F_{X}\left(t\right)}\right)|X>t\right], \end{array}$$

*where $g_{t}\left(v\right)=0$ for $v\in [0,F_{X}\left(t\right))$, and $g_{t}\left(v\right)=\left(\frac{1-v}{1-F_{X}\left(t\right)}\right){\left[-\log\left(\frac{1-v}{1-F_{X}\left(t\right)}\right)\right]}^{\alpha }$ for $v\in [F_{X}\left(t\right),1]$.*

**Example** **13.** *Let $g\left(t\right)=\frac{1}{\alpha -1}\left[\left(1-t\right)-{\left(1-t\right)}^{\alpha }\right],\;\alpha >0,\;\alpha \neq 1,\;t\in \left[0,1\right]$, in Corollary 5, Equation (12) denoted by ${\mathrm{DCRT}}_{\alpha ,t}\left(X\right)$; we have*

$$\begin{array}{cc} {\mathrm{DCRT}}_{\alpha ,t}\left(X\right)\; & =\int_{t}^{+\infty }\frac{1}{\alpha -1}\left[\frac{{\bar{F}}_{X}\left(x\right)}{{\bar{F}}_{X}\left(t\right)}-{\left(\frac{{\bar{F}}_{X}\left(x\right)}{{\bar{F}}_{X}\left(t\right)}\right)}^{\alpha }\right]dx=-\int_{0}^{1}F_{X}^{-1}\left(v\right)dg_{t}\left(v\right)=-\frac{\alpha }{\alpha -1}\mathrm{Cov}\left[X,{\left(\frac{{\bar{F}}_{X}\left(X\right)}{{\bar{F}}_{X}\left(t\right)}\right)}^{\alpha -1}|X>t\right], \end{array}$$

*where $g_{t}\left(v\right)=0$ for $v\in [0,F_{X}\left(t\right))$, and $g_{t}\left(v\right)=\frac{1}{\alpha -1}\left[\frac{1-v}{1-F_{X}\left(t\right)}-{\left(\frac{1-v}{1-F_{X}\left(t\right)}\right)}^{\alpha }\right]$ for $v\in [F_{X}\left(t\right),1]$.*

In particular, when $X\in L_{+}^{0}$, the above measure is the dynamic cumulative residual Tsallis entropy of order α introduced by Rajesh and Sunoj [13].

In particular, when $X\in L_{+}^{0}$ and α=2, we obtain (dynamic Gini mean semi-difference)

$$\begin{array}{cc} {\mathrm{DCRT}}_{2,t}\left(X\right)\; & =\int_{t}^{+\infty }\left[\frac{{\bar{F}}_{X}\left(x\right)}{{\bar{F}}_{X}\left(t\right)}-{\left(\frac{{\bar{F}}_{X}\left(x\right)}{{\bar{F}}_{X}\left(t\right)}\right)}^{2}\right]dx=-\int_{0}^{1}F_{X}^{-1}\left(v\right)dg_{t}\left(v\right)=-2\mathrm{Cov}\left[X,\frac{{\bar{F}}_{X}\left(X\right)}{{\bar{F}}_{X}\left(t\right)}|X>t\right], \end{array}$$

where $g_{t}\left(v\right)=0$ for $v\in [0,F_{X}\left(t\right))$, and $g_{t}\left(v\right)=\left[\frac{1-v}{1-F_{X}\left(t\right)}-{\left(\frac{1-v}{1-F_{X}\left(t\right)}\right)}^{2}\right]$ for $v\in [F_{X}\left(t\right),1]$.

**Example** **14.** *Let $g\left(t\right)={\left(1-t\right)}^{1/2}-\left(1-t\right),\;t\in \left[0,1\right]$, in Corollary 5; we have*

$$\begin{array}{c} \int_{t}^{+\infty }\left[{\left(\frac{{\bar{F}}_{X}\left(x\right)}{{\bar{F}}_{X}\left(t\right)}\right)}^{1/2}-\frac{{\bar{F}}_{X}\left(x\right)}{{\bar{F}}_{X}\left(t\right)}\right]dx=-\int_{0}^{1}F_{X}^{-1}\left(u\right)dg_{t}\left(u\right)=\frac{1}{2}\mathrm{Cov}\left[X,\frac{{\bar{F}}_{X}\left(X\right)}{{\bar{F}}_{X}\left(t\right)}|X>t\right], \end{array}$$

*where $g_{t}\left(v\right)=0$ for $v\in [0,F_{X}\left(t\right))$, and $g_{t}\left(v\right)={\left(\frac{1-v}{1-F_{X}\left(t\right)}\right)}^{1/2}-\frac{1-v}{1-F_{X}\left(t\right)}$ for $v\in [F_{X}\left(t\right),1]$.*

**Example** **15.** *Let $g\left(t\right)=-2[t+{\left(1-t\right)}^{r}-1],\;r>1,\;t\in \left[0,1\right]$, in Corollary 5; we have*

$$\begin{array}{c} -\int_{t}^{+\infty }2\left[\frac{F_{X}\left(x\right)-F_{X}\left(t\right)}{{\bar{F}}_{X}\left(t\right)}+{\left(\frac{{\bar{F}}_{X}\left(x\right)}{{\bar{F}}_{X}\left(t\right)}\right)}^{r}-1\right]dx=\int_{0}^{1}F_{X}^{-1}\left(u\right)dg_{t}\left(u\right)=-2r\mathrm{Cov}\left[X,{\left(\frac{{\bar{F}}_{X}\left(X\right)}{{\bar{F}}_{X}\left(t\right)}\right)}^{r-1}|X>t\right], \end{array}$$

*where $g_{t}\left(v\right)=0$ for $v\in [0,F_{X}\left(t\right))$, and $g_{t}\left(v\right)=2\left[\frac{v-F_{X}\left(t\right)}{1-F_{X}\left(t\right)}+{\left(\frac{1-v}{1-F_{X}\left(t\right)}\right)}^{r}-1\right]$ for $v\in [F_{X}\left(t\right),1]$.*

**Example** **16.** *Let $g\left(t\right)=\frac{1}{n!}\left(1-t\right){\left[-\log\left(1-t\right)\right]}^{n},\;n\in \left\{1,2,\ldots \right\},\;t\in \left[0,1\right]$ in Corollary 4, Equation (11) denoted by ${\mathrm{DGCRE}}_{n,t}\left(X\right)$, so that $l\left(t\right)=\frac{1}{n!}{\left[-\log\left(1-t\right)\right]}^{n-1}\left[n+\log\left(1-t\right)\right]$. Then, we have*

$$\begin{array}{cc} {\mathrm{DGCRE}}_{n,t}\left(X\right)\; & =\frac{1}{n!}\int_{t}^{+\infty }\frac{{\bar{F}}_{X}\left(x\right)}{{\bar{F}}_{X}\left(t\right)}{\left[-\log\left(\frac{{\bar{F}}_{X}\left(x\right)}{{\bar{F}}_{X}\left(t\right)}\right)\right]}^{n}dx=-\int_{0}^{1}F_{X}^{-1}\left(u\right)dg_{t}\left(u\right)=-\mathrm{Cov}\left[X,l\left(\frac{F_{X}\left(X\right)-F_{X}\left(t\right)}{1-F_{X}\left(t\right)}\right)|X>t\right], \end{array}$$

*where $g_{t}\left(v\right)=0$ for $v\in [0,F_{X}\left(t\right))$, and $g_{t}\left(v\right)=\frac{1}{n!}\left(\frac{1-v}{1-F_{X}\left(t\right)}\right){\left[-\log\left(\frac{1-v}{1-F_{X}\left(t\right)}\right)\right]}^{n}$ for $v\in [F_{X}\left(t\right),1]$.*

In particular, when $X\in L_{+}^{0}$, the above measure denotes the dynamic generalized cumulative residual entropy introduced by Psarrakos and Navarro [18].

**Example** **17.** *Let $g\left(t\right)=\frac{1}{n!}\left(1-t\right){\left[-\log\left(1-t\right)\right]}^{n},\;t\in \left[0,1\right]$ in Theorem 3, Equation (10) denoted by ${\mathrm{DWGCRE}}_{n,\psi ,t}\left(X\right)$, so that $l\left(t\right)=\frac{1}{n!}{\left[-\log\left(1-t\right)\right]}^{n-1}\left[n+\log\left(1-t\right)\right]$. Then, we have*

$$\begin{array}{cc} {\mathrm{DWGCRE}}_{n,\psi ,t}\left(X\right)\; & =\frac{1}{n!}\int_{t}^{+\infty }\psi \left(x\right){\bar{F}}_{X}\left(x\right){\left[-\log\left({\bar{F}}_{X}\left(x\right)\right)\right]}^{n}dx=-\int_{0}^{1}\Psi \left(F_{X}^{-1}\left(u\right)\right)dg_{t}\left(u\right)\\ \; & =-\mathrm{Cov}\left[\Psi \left(X\right),l\left(\frac{F_{X}\left(X\right)-F_{X}\left(t\right)}{1-F_{X}\left(t\right)}\right)|X>t\right], \end{array}$$

*where $g_{t}\left(v\right)=0$ for $v\in [0,F_{X}\left(t\right))$, and $g_{t}\left(v\right)=\frac{1}{n!}\left(\frac{1-v}{1-F_{X}\left(t\right)}\right){\left[-\log\left(\frac{1-v}{1-F_{X}\left(t\right)}\right)\right]}^{n}$ for $v\in [F_{X}\left(t\right),1]$.*

In particular, when $X\in L_{+}^{0}$, the above measure is the dynamic WGCRE introduced by Tahmasebi [25].

Particularly, when n=1 and ψ(x)=x, the ${\mathrm{DWGCRE}}_{n,\psi ,t}\left(X\right)$ reduces to the dynamic weighted cumulative residual entropy (${\mathrm{DWCRE}}_{t}\left(X\right)$) defined as

$$\begin{array}{cc} {\mathrm{DWCRE}}_{t}\left(X\right)\; & =\int_{t}^{+\infty }x{\bar{F}}_{X}\left(x\right)\left[-\log\left({\bar{F}}_{X}\left(x\right)\right)\right]dx=-\frac{1}{2}\int_{0}^{1}{\left(F_{X}^{-1}\left(u\right)\right)}^{2}dg_{t}\left(u\right)=-\frac{1}{2}\mathrm{Cov}\left[X^{2},\log\left({\bar{F}}_{X}\left(X\right)\right)|X>t\right], \end{array}$$

where $g_{t}\left(v\right)=0$ for $v\in [0,F_{X}\left(t\right))$, and $g_{t}\left(v\right)=\left(\frac{1-v}{1-F_{X}\left(t\right)}\right)\left[-\log\left(\frac{1-v}{1-F_{X}\left(t\right)}\right)\right]$ for $v\in [F_{X}\left(t\right),1]$. As a special case, when $X\in L_{+}^{0}$, the above measure is introduced by Miralia and Baratpour [30]. They also defined ${\mathrm{DWGCRE}}_{n,\psi ,t}\left(X\right)$, n=1, i.e.,

$$\begin{array}{cc} {\mathrm{DWGCRE}}_{\psi ,t}\left(X\right)\; & =\int_{t}^{+\infty }\psi \left(x\right){\bar{F}}_{X}\left(x\right)\left[-\log\left({\bar{F}}_{X}\left(x\right)\right)\right]dx=-\int_{0}^{1}\Psi \left(F_{X}^{-1}\left(u\right)\right)dg_{t}\left(u\right)=-\mathrm{Cov}\left[\Psi \left(X\right),\log\left({\bar{F}}_{X}\left(X\right)\right)|X>t\right], \end{array}$$

where $g_{t}\left(v\right)=0$ for $v\in [0,F_{X}\left(t\right))$, and $g_{t}\left(v\right)=\left(\frac{1-v}{1-F_{X}\left(t\right)}\right)\left[-\log\left(\frac{1-v}{1-F_{X}\left(t\right)}\right)\right]$ for $v\in [F_{X}\left(t\right),1]$.

**Example** **18.** *Let $g\left(t\right)=\frac{1}{\alpha -1}\left(t-t^{\alpha }\right),\;\alpha >0,\;\alpha \neq 1,\;t\in \left[0,1\right]$ in Corollary 7, Equation (16) denoted by ${\mathrm{DCT}}_{\alpha ,(t)}\left(X\right)$; we have*

$$\begin{array}{cc} {\mathrm{DCT}}_{\alpha ,(t)}\left(X\right)\; & =\int_{-\infty }^{t}\frac{1}{\alpha -1}\left[\frac{F_{X}\left(x\right)}{F_{X}\left(t\right)}-{\left(\frac{F_{X}\left(x\right)}{F_{X}\left(t\right)}\right)}^{\alpha }\right]dx=-\int_{0}^{1}F_{X}^{-1}\left(u\right)dg_{\left(t\right)}\left(u\right)=\frac{\alpha }{\alpha -1}\mathrm{Cov}\left[X,{\left(\frac{F_{X}\left(X\right)}{F_{X}\left(t\right)}\right)}^{\alpha -1}|X<t\right], \end{array}$$

*where $g_{\left(t\right)}\left(v\right)=0$ for $v\in (F_{X}\left(t\right),1]$, and $g_{\left(t\right)}\left(v\right)=\frac{1}{\alpha -1}\left[\frac{v}{F_{X}\left(t\right)}-{\left(\frac{v}{F_{X}\left(t\right)}\right)}^{\alpha }\right]$ for $v\in [0,F_{X}\left(t\right)]$.*

In particular, when n=1 and $X\in L_{+}^{0}$, the above measure is a generalization of the dynamic cumulative Tsallis entropy introduced by Calì et al. [10]. Note that when α→1, it reduces to (a generalization of) cumulative past entropy, defined as (see, e.g., [31])

$$\begin{array}{cc} CE_{\left(t\right)}\left(X\right)\; & =-\int_{0}^{t}\frac{F_{X}\left(x\right)}{F_{X}\left(t\right)}\log\left(\frac{F_{X}\left(x\right)}{F_{X}\left(t\right)}\right)dx=\int_{0}^{1}F_{X}^{-1}\left(v\right)dh_{\left(t\right)}\left(v\right)=\mathrm{Cov}\left[X,\log\left(F_{X}\left(X\right)\right)|X<t\right], \end{array}$$

where $h_{\left(t\right)}\left(v\right)=0$ for $v\in (F_{X}\left(t\right),1]$, and $h_{\left(t\right)}\left(v\right)=\frac{v}{F_{X}\left(t\right)}\log\left(\frac{v}{F_{X}\left(t\right)}\right)$ for $v\in [0,F_{X}\left(t\right)]$.

**Example** **19.** *Let $g\left(t\right)=\frac{1}{n!}t{\left[-\log t\right]}^{n},\;n\in \left\{1,2,\ldots \right\},\;t\in \left[0,1\right]$, in Corollary 6, Equation (15) denoted by $DGCE_{n,(t)}\left(X\right)$, so that $l\left(t\right)=\frac{1}{n!}{\left[-\log t\right]}^{n-1}\left[n+\log t\right]$. Then, we have*

$$\begin{array}{cc} DGCE_{n,(t)}\left(X\right)\; & =\frac{1}{n!}\int_{-\infty }^{t}\frac{F_{X}\left(x\right)}{F_{X}\left(t\right)}{\left[-\log\left(\frac{F_{X}\left(x\right)}{F_{X}\left(t\right)}\right)\right]}^{n}dx=-\int_{0}^{1}F_{X}^{-1}\left(u\right)dg_{\left(t\right)}\left(u\right)=\mathrm{Cov}\left[X,l\left(\frac{F_{X}\left(X\right)}{F_{X}\left(t\right)}\right)|X<t\right], \end{array}$$

*where $g_{\left(t\right)}\left(v\right)=0$ for $v\in (F_{X}\left(t\right),1]$, and $g_{t}\left(v\right)=\frac{1}{n!}\left(\frac{v}{F_{X}\left(t\right)}\right){\left[-\log\left(\frac{v}{F_{X}\left(t\right)}\right)\right]}^{n}$ for $v\in [0,F_{X}\left(t\right)]$.*

In particular, when $X\in L_{+}^{0}$, the above measure is the dynamic generalized cumulative entropy introduced by Kayal [19].

**Example** **20.** *Let $g\left(t\right)=\frac{1}{n!}t{\left[-\log t\right]}^{n},\;n\in \left\{1,2,\ldots \right\},\;t\in \left[0,1\right]$ in Theorem 4, Equation (14) denoted by $DGCE_{n,\psi ,(t)}\left(X\right)$, so that $l\left(t\right)=\frac{1}{n!}{\left[-\log t\right]}^{n-1}\left[n+\log t\right]$. Then, we have*

$$\begin{array}{cc} DGCE_{n,\psi ,(t)}\left(X\right)\; & =\frac{1}{n!}\int_{-\infty }^{t}\psi \left(x\right)\frac{F_{X}\left(x\right)}{F_{X}\left(t\right)}{\left[-\log\left(\frac{F_{X}\left(x\right)}{F_{X}\left(t\right)}\right)\right]}^{n}dx=-\int_{0}^{1}\Psi \left(F_{X}^{-1}\left(u\right)\right)dg_{\left(t\right)}\left(u\right)=\mathrm{Cov}\left[\Psi \left(X\right),l\left(\frac{F_{X}\left(X\right)}{F_{X}\left(t\right)}\right)|X<t\right], \end{array}$$

*where $g_{\left(t\right)}\left(v\right)=0$ for $v\in (F_{X}\left(t\right),1]$, and $g_{t}\left(v\right)=\frac{1}{n!}\left(\frac{v}{F_{X}\left(t\right)}\right){\left[-\log\left(\frac{v}{F_{X}\left(t\right)}\right)\right]}^{n}$ for $v\in [0,F_{X}\left(t\right)]$.*

In particular, when n=1 and $X\in L_{+}^{0}$, $DGCE_{n,\psi ,(t)}\left(X\right)$ is reduced as (see [30])

$$\begin{array}{cc} DGCE_{\psi ,(t)}\left(X\right)\; & =\int_{0}^{t}\psi \left(x\right)\frac{F_{X}\left(x\right)}{F_{X}\left(t\right)}\left[-\log\left(\frac{F_{X}\left(x\right)}{F_{X}\left(t\right)}\right)\right]dx=-\int_{0}^{1}\Psi \left(F_{X}^{-1}\left(u\right)\right)dh_{\left(t\right)}\left(u\right)=\mathrm{Cov}\left[\Psi \left(X\right),\log\left(F_{X}\left(X\right)\right)|X<t\right], \end{array}$$

where $h_{\left(t\right)}\left(v\right)=0$ for $v\in (F_{X}\left(t\right),1]$, and $h_{t}\left(v\right)=\left(\frac{v}{F_{X}\left(t\right)}\right)\left[-\log\left(\frac{v}{F_{X}\left(t\right)}\right)\right]$ for $v\in [0,F_{X}\left(t\right)]$.

### 2.5. Discussion

Note that the above entropy risk measures satisfy (B1) standardization by their covariance representations. For any c∈R, using $F_{X+c}^{-1}\left(u\right)=F_{X}^{-1}\left(u\right)+c$, we obtain that initial entropy and simple dynamic entropy risk measures satisfy (B2) location invariance, but weighted entropy risk measures do not satisfy (B2). Therefore, initial entropy and simple dynamic entropy risk measures are measures of variability.

For any $\lambda \in R_{+}$, using $F_{\lambda X}^{-1}\left(u\right)=\lambda F_{X}^{-1}\left(u\right)$, we obtain that initial entropy and simple dynamic entropy risk measures satisfy (B3) positive homogeneity.

When g:[0,1]→R is finite variation and g(1)=g(0)=0, the signed Choquet integral is defined by

(20) $$\begin{array}{c} I\left(X\right)=-\int_{-\infty }^{\infty }g\left(F_{X}\left(x\right)\right)dx,\;\mathrm{for}\;\mathrm{all}\;X\in \chi . \end{array}$$

When *g* is right-continuous, then Equation (20) can be expressed as

(21) $$\begin{array}{c} I\left(X\right)=\int_{0}^{1}F_{X}^{-1}\left(s\right)dg\left(s\right). \end{array}$$

Furthermore, when g is absolutely continuous, with dg(s)=l(s)ds, then Equation (21) can be expressed as

(22) $$\begin{array}{c} I\left(X\right)=\int_{0}^{1}F_{X}^{-1}\left(s\right)l\left(s\right)ds. \end{array}$$

From (21), we can see that the signed Choquet integral satisfies the co-monotonically additive property ([32]). Thus, initial entropy and simple dynamic entropy risk measures are co-monotonically additive measures of variability.

The functional I(·) defined by Equation (20) is sub-additive if and only if g is convex (e.g., [33,34]). Hence, initial entropy risk measures, which are shown in Examples 1–5 and (17)–(19), satisfy (A4) sub-additivity. Therefore, Examples 1–5 and (17)–(19) are co-monotonically additive and coherent measures of variability.

These initial entropy risk measures can be applied to the predictability of the failure time of a system (see [11,14]). The weighted entropy risk measures are shift-dependent measures of uncertainty, and can be applied to some practical situations of reliability and neurobiology (see [35,36]). The dynamic entropy risk measures can be used to capture effects of the age *t* of an individual or an item under study on the information about the residual lifetime (see [29]).

**Figure 1:**
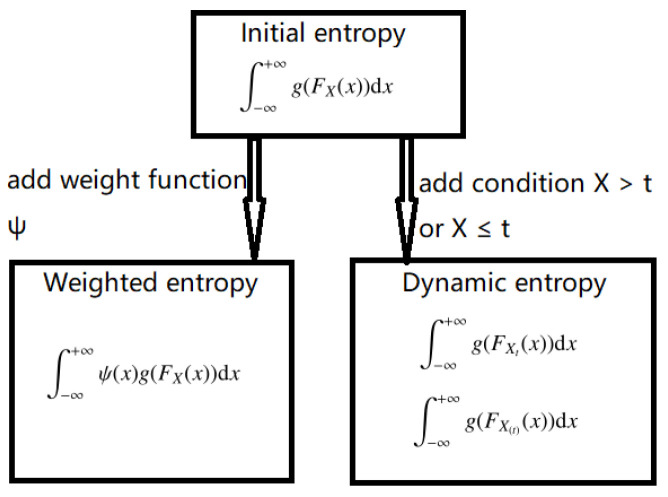
The relationship between three entropy risk measures.

The initial, weighted and dynamic entropy risk measures are closely related, as shown in Figure 1:

From a risk measure point of view, the initial entropy risk measures can capture the variability of a financial position as a whole. The dynamic entropy risk measures can depict the variability of a financial position focused on feasible choices of the ranges (upper tail or lower tail).

**Figure 2:**
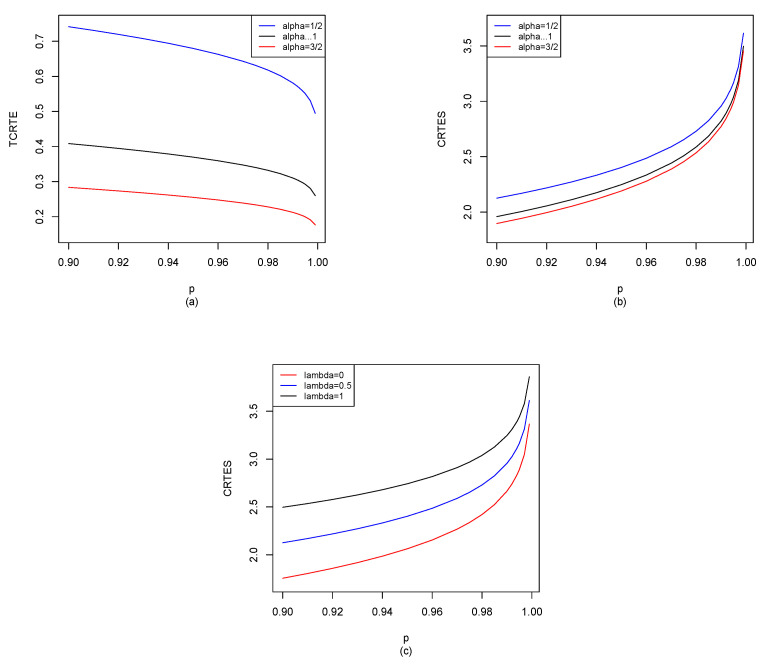
$N_{1}\left(0,\;1\right)$: (**a**) ${\mathrm{TCRTE}}_{\alpha ,p}\left(X\right)$ of $\alpha =\frac{1}{2}$, α→1 and $\alpha =\frac{3}{2}$; (**b**) ${\mathrm{CRTES}}_{\alpha ,p}^{\lambda }\left(X\right)$ of $\alpha =\frac{1}{2}$, α→1 and $\alpha =\frac{3}{2}$ with λ=0.5; (**c**) ${\mathrm{CRTES}}_{\alpha ,p}^{\lambda }\left(X\right)$ of λ=0, λ=0.5 and λ=1 with $\alpha =\frac{1}{2}$.

**Figure 3:**
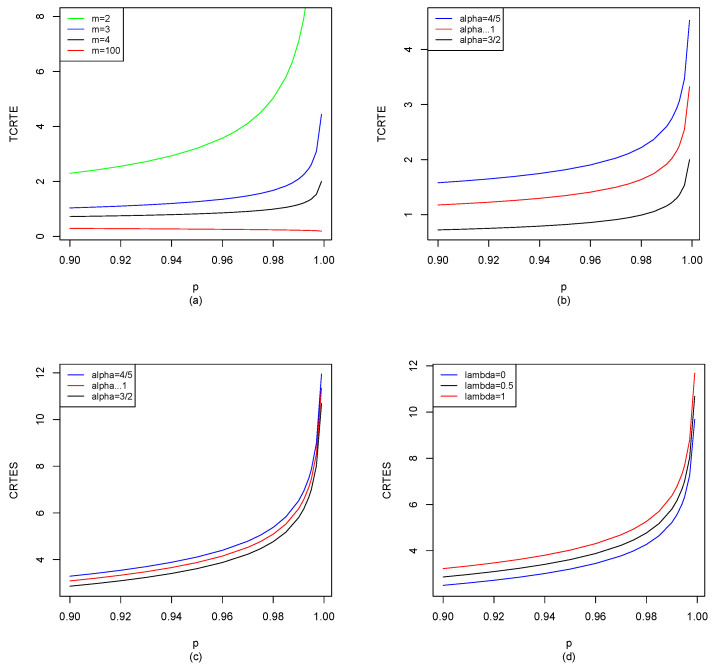
$St_{1}\left(0,\;1,\;m\right)$: (**a**) ${\mathrm{TCRTE}}_{\alpha ,p}\left(X\right)$ of m=2, m=3, m=4 and m=100 with $\alpha =\frac{3}{2}$; (**b**) ${\mathrm{TCRTE}}_{\alpha ,p}\left(X\right)$ of $\alpha =\frac{4}{5}$, α→1 and $\alpha =\frac{3}{2}$ with m=4; (**c**) ${\mathrm{CRTES}}_{\alpha ,p}^{\lambda }\left(X\right)$ of $\alpha =\frac{4}{5}$, α→1 and $\alpha =\frac{3}{2}$ with m=4 and λ=0.5; (**d**) ${\mathrm{CRTES}}_{\alpha ,p}^{\lambda }\left(X\right)$ of λ=0, λ=0.5 and λ=1 with m=4 and $\alpha =\frac{3}{2}$.

**Figure 4:**
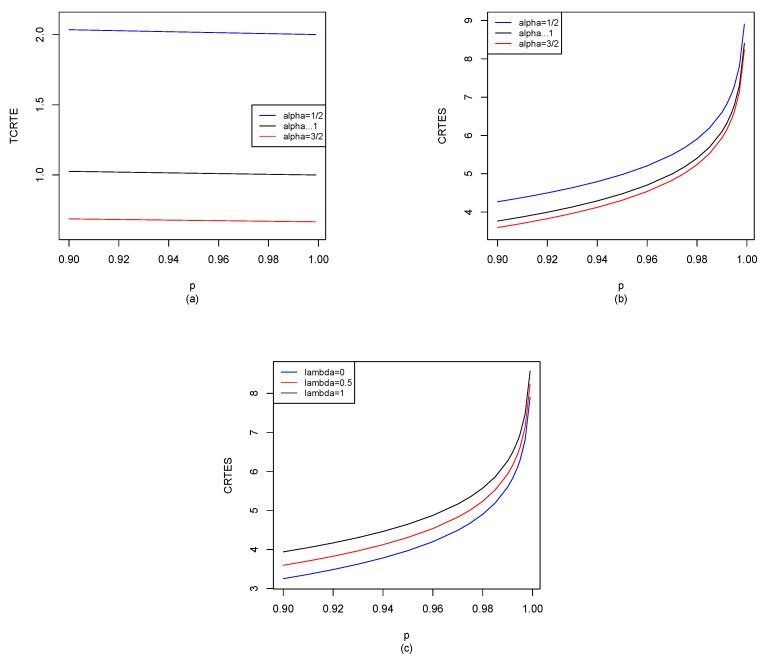
$Lo_{1}\left(0,\;1\right)$: (**a**) ${\mathrm{TCRTE}}_{\alpha ,p}\left(X\right)$ of $\alpha =\frac{1}{2}$, α→1 and $\alpha =\frac{3}{2}$; (**b**) ${\mathrm{CRTES}}_{\alpha ,p}^{\lambda }\left(X\right)$ of $\alpha =\frac{1}{2}$, α→1 and $\alpha =\frac{3}{2}$ with λ=0.5; (**c**) ${\mathrm{CRTES}}_{\alpha ,p}^{\lambda }\left(X\right)$ of λ=0, λ=0.5 and λ=1 with $\alpha =\frac{3}{2}$.

**Figure 5:**
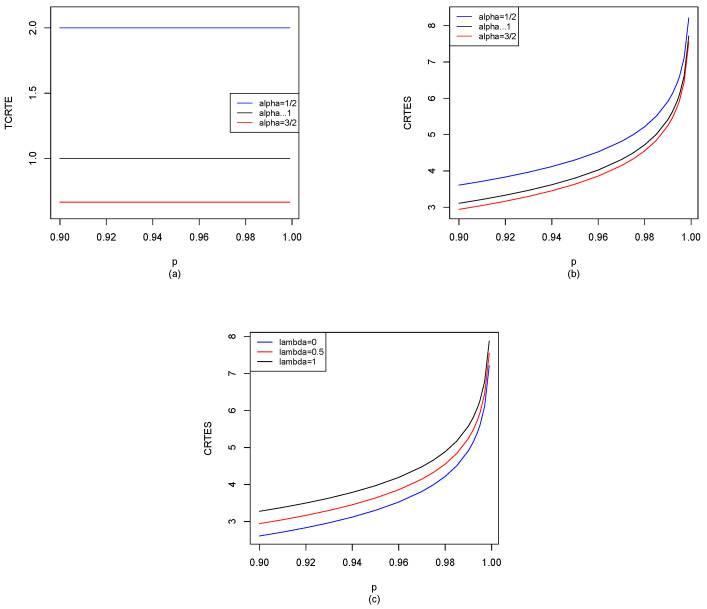
$La_{1}\left(0,\;1\right)$: (**a**) ${\mathrm{TCRTE}}_{\alpha ,p}\left(X\right)$ of $\alpha =\frac{1}{2}$, α→1 and $\alpha =\frac{3}{2}$; (**b**) ${\mathrm{CRTES}}_{\alpha ,p}^{\lambda }\left(X\right)$ of $\alpha =\frac{1}{2}$, α→1 and $\alpha =\frac{3}{2}$ with λ=0.5; (**c**) ${\mathrm{CRTES}}_{\alpha ,p}^{\lambda }\left(X\right)$ of λ=0, λ=0.5 and λ=1 with $\alpha =\frac{3}{2}$.

**Figure 6:**
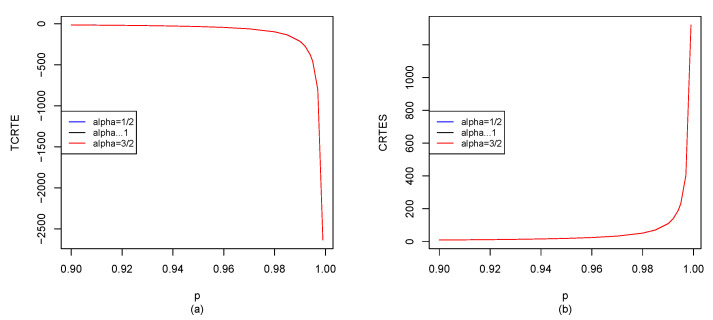
IG(10,1): (**a**) ${\mathrm{TCRTE}}_{\alpha ,p}\left(X\right)$ of $\alpha =\frac{1}{2}$, α→1 and $\alpha =\frac{3}{2}$; (**b**) ${\mathrm{CRTES}}_{\alpha ,p}^{\lambda }\left(X\right)$ of $\alpha =\frac{1}{2}$, α→1 and $\alpha =\frac{3}{2}$ with λ=0.5.

**Figure 7:**
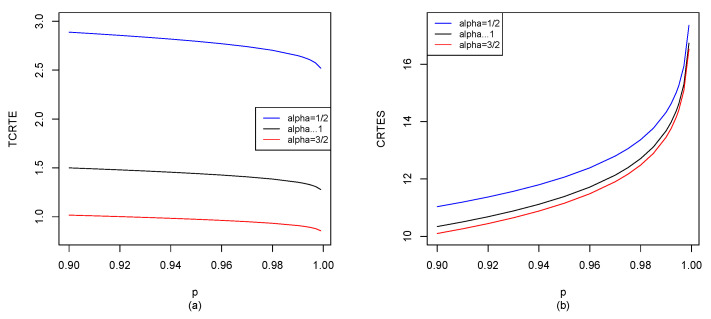
Γ(5,1): (**a**) ${\mathrm{TCRTE}}_{\alpha ,p}\left(X\right)$ of $\alpha =\frac{1}{2}$, α→1 and $\alpha =\frac{3}{2}$; (**b**) ${\mathrm{CRTES}}_{\alpha ,p}^{\lambda }\left(X\right)$ of $\alpha =\frac{1}{2}$, α→1 and $\alpha =\frac{3}{2}$ with λ=0.5.

**Figure 8:**
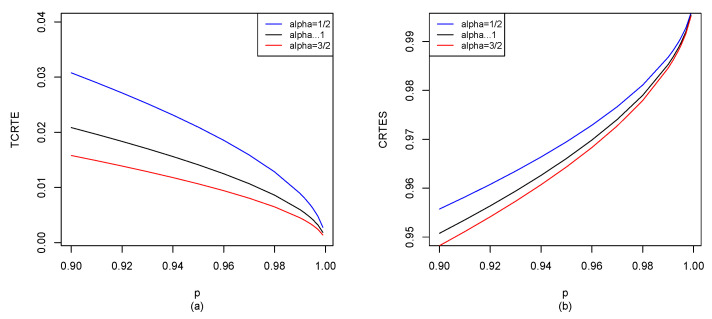
B(5,2): (**a**) ${\mathrm{TCRTE}}_{\alpha ,p}\left(X\right)$ of $\alpha =\frac{1}{2}$, α→1 and $\alpha =\frac{3}{2}$; (**b**) ${\mathrm{CRTES}}_{\alpha ,p}^{\lambda }\left(X\right)$ of $\alpha =\frac{1}{2}$, α→1 and $\alpha =\frac{3}{2}$ with λ=0.5.

In finance and risk management, Markowitz’s mean-variance portfolio theory plays a vital role in modern portfolio theory. It is known that the initial entropy and simple dynamic entropy risk measures are measures of variability. We can replace variance with the initial entropy and simple dynamic entropy risk measures, respectively. The initial entropy risk measure is used to capture ordinary (general) risk, and it is favored by investors, such as the firm’s ordinary business and the shareholders’ interests. The dynamic entropy risk measure is used to depict the tails of risks (extreme events), which is to reduce (or avoid) the impact of extreme events and is favored by regulators and decision makers (see [37]). For example, we give ${\mathrm{CRTES}}_{\alpha ,p}^{\lambda }\left(X\right)$ for different distributions in Section 5, and also use the R software to compute ${\mathrm{CRTES}}_{\alpha ,p}^{\lambda }\left(X\right)$ for p∈[0.9,1), shown in Figure 2, Figure 3, Figure 4, Figure 5, Figure 6, Figure 7 and Figure 8. When α→1, ${\mathrm{CRTES}}_{\alpha ,p}^{\lambda }\left(X\right)$ reduces to ${\mathrm{TCRE}}_{p}\left(X\right)$ given by Hu and Chen [6], we can observe the difference between our results and Hu and Chen’s results through Figure 2, Figure 3, Figure 4, Figure 5, Figure 6, Figure 7 and Figure 8. Other potential applications of these entropy risk measures need to be further explored in the future.

## 3. Tail-Based Entropies

Let $t=x_{p}$ in Example 13; we obtain tail-based cumulative residual Tsallis entropy of order α:

(23) $$\begin{array}{c} {\mathrm{TCRTE}}_{\alpha ,p}\left(X\right)=\int_{x_{p}}^{+\infty }\frac{1}{\alpha -1}\left[\frac{{\bar{F}}_{X}\left(x\right)}{1-p}-{\left(\frac{{\bar{F}}_{X}\left(x\right)}{1-p}\right)}^{\alpha }\right]dx=-\int_{0}^{1}F_{X}^{-1}\left(s\right)dg_{p}\left(s\right)=-\frac{\alpha }{\alpha -1}\mathrm{Cov}\left[X,{\left(\frac{{\bar{F}}_{X}\left(X\right)}{1-p}\right)}^{\alpha -1}|X>x_{p}\right], \end{array}$$

where $g_{p}\left(s\right)=0$ for s∈[0,p), and $g_{p}\left(s\right)=\frac{1}{\alpha -1}\left[\frac{1-s}{1-p}-{\left(\frac{1-s}{1-p}\right)}^{\alpha }\right]$ for s∈[p,1].

Let $t=x_{p}$ in Example 12; in this case, $l\left(t\right)={\left[-\log\left(1-t\right)\right]}^{\alpha -1}\left[\alpha +\log\left(1-t\right)\right]$, 0<α≤1 and we obtain tail-based fractional cumulative residual entropy:

(24) $$\begin{array}{c} {\mathrm{TFCRE}}_{\alpha ,p}\left(X\right)=\int_{x_{p}}^{+\infty }\frac{{\bar{F}}_{X}\left(x\right)}{1-p}{\left[-\log\left(\frac{{\bar{F}}_{X}\left(x\right)}{1-p}\right)\right]}^{\alpha }dx=-\int_{0}^{1}F_{X}^{-1}\left(s\right)dg_{p}\left(s\right)=-\mathrm{Cov}\left[X,l\left(\frac{F_{X}\left(X\right)-p}{1-p}\right)|X>x_{p}\right], \end{array}$$

where $g_{p}\left(s\right)=0$ for s∈[0,p), and $g_{p}\left(s\right)=\left(\frac{1-s}{1-p}\right){\left[-\log\left(\frac{1-s}{1-p}\right)\right]}^{\alpha }$ for s∈[p,1].

**Remark** **2.** *Let α=1 in (24) (or α→1 in (23)); we obtain the tail-based cumulative residual entropy (${\mathrm{TCRE}}_{p}\left(X\right)$) given by Hu and Chen [6]:*

$$\begin{array}{cc} {\mathrm{TCRE}}_{p}\left(X\right)\; & =-\int_{x_{p}}^{+\infty }\frac{{\bar{F}}_{X}\left(x\right)}{1-p}\log\left(\frac{{\bar{F}}_{X}\left(x\right)}{1-p}\right)dx=\int_{0}^{1}F_{X}^{-1}\left(s\right)dh_{x_{p}}\left(s\right)=-\mathrm{Cov}\left[X,\log\left({\bar{F}}_{X}\left(X\right)\right)|X>x_{p}\right], \end{array}$$

*where $h_{x_{p}}\left(s\right)=0$ for s∈[0,p), and $h_{x_{p}}\left(s\right)=\left(\frac{1-s}{1-p}\right)\log\left(\frac{1-s}{1-p}\right)$ for s∈[p,1].*

Let $t=x_{p}$ in Example 14; we obtain tail-based right-tail deviation:

$$\begin{array}{c} {\mathrm{TRTD}}_{p}\left(X\right)=\int_{x_{p}}^{+\infty }\left[{\left(\frac{{\bar{F}}_{X}\left(x\right)}{1-p}\right)}^{1/2}-\frac{{\bar{F}}_{X}\left(x\right)}{1-p}\right]dx=-\int_{0}^{1}F_{X}^{-1}\left(s\right)dg_{p}\left(s\right)=\frac{1}{2}\mathrm{Cov}\left[X,\frac{{\bar{F}}_{X}\left(X\right)}{1-p}|X>x_{p}\right], \end{array}$$

where $g_{p}\left(s\right)=0$ for s∈[0,p), and $g_{p}\left(s\right)={\left(\frac{1-s}{1-p}\right)}^{1/2}-\frac{1-s}{1-p}$ for s∈[p,1].

**Remark** **3.** *Let $\alpha =\frac{1}{2}$ in (23); we observe that*

(25) $$\begin{array}{c} {\mathrm{TRTD}}_{p}\left(X\right)=\frac{1}{2}{\mathrm{TCRTE}}_{\frac{1}{2},p}\left(X\right). \end{array}$$

Let $t=x_{p}$ in Example 15; we obtain the tail-based extended Gini coefficient (see [7]):

(26) $$\begin{array}{c} {\mathrm{TEGini}}_{r,p}=-\int_{x_{p}}^{+\infty }2\left[\frac{F_{X}\left(x\right)-p}{1-p}+{\left(\frac{{\bar{F}}_{X}\left(x\right)}{1-p}\right)}^{r}-1\right]dx=\int_{0}^{1}F_{X}^{-1}\left(s\right)dg_{p}\left(s\right)=-2r\mathrm{Cov}\left[X,{\left(\frac{{\bar{F}}_{X}\left(X\right)}{1-p}\right)}^{r-1}|X>x_{p}\right], \end{array}$$

where $g_{p}\left(s\right)=0$ for s∈[0,p), and $g_{p}\left(s\right)=2\left[\frac{s-p}{1-p}+{\left(\frac{1-s}{1-p}\right)}^{r}-1\right]$ for s∈[p,1].

**Remark** **4.** *Let r=2 in (26); we obtain tail Gini (see [5]):*

$$\begin{array}{cc} {\mathrm{TGini}}_{p}\left(X\right)\; & =2\int_{x_{p}}^{+\infty }\left[\frac{{\bar{F}}_{X}\left(x\right)}{1-p}-{\left(\frac{{\bar{F}}_{X}\left(x\right)}{1-p}\right)}^{2}\right]dx=\int_{0}^{1}F_{X}^{-1}\left(s\right)dg_{p}\left(s\right)=-4\mathrm{Cov}\left[X,\frac{{\bar{F}}_{X}\left(X\right)}{1-p}|X>x_{p}\right], \end{array}$$

*where $g_{p}\left(s\right)=0$ for s∈[0,p), and $g_{p}\left(s\right)=2\left[\frac{s-1}{1-p}+{\left(\frac{1-s}{1-p}\right)}^{2}\right]$ for s∈[p,1].*

## 4. Shortfalls of Entropy

We now introduce two risk measures of entropy shortfall, which are linear combinations of ${\mathrm{ES}}_{p}$, ${\mathrm{TCRTE}}_{\alpha ,p}$ and ${\mathrm{TRTD}}_{p}$, respectively:

$$\begin{array}{c} {\mathrm{CRTES}}_{\alpha ,p}^{\lambda }\left(X\right)={\mathrm{ES}}_{p}\left(X\right)+\lambda \cdot {\mathrm{TCRTE}}_{\alpha ,p}\left(X\right), \end{array}$$

$$\begin{array}{c} {\mathrm{RTDS}}_{p}^{\lambda }\left(X\right)={\mathrm{ES}}_{p}\left(X\right)+\lambda \cdot {\mathrm{TRTD}}_{p}\left(X\right), \end{array}$$

where p∈[0,1) is also a confidence level, and λ≥0 is a loading parameter.

**Theorem** **5.** *Assume that p∈(0,1), λ∈[0,∞) and the convex cone $X=L^{2}$.*

*(1)*  *${\mathrm{CRTES}}_{\alpha ,p}^{\lambda }$ is represented as*
(27) $$\begin{array}{cc} {\mathrm{CRTES}}_{\alpha ,p}^{\lambda }\left(X\right) & =\int_{0}^{1}F_{X}^{-1}\left(v\right)dg_{p,\lambda }\left(v\right) \end{array}$$
(28) $$\begin{array}{cc} \; & =\int_{0}^{1}F_{X}^{-1}\left(v\right)\varpi_{p,\lambda }\left(v\right)dv, \end{array}$$
  *where*
$$\begin{array}{cc} g_{p,\lambda }\left(v\right) & =\frac{1}{1-p}{\left(v-p\right)}_{+}-\frac{\lambda }{\alpha -1}\cdot \left[\frac{1-v}{1-p}-{\left(\frac{1-v}{1-p}\right)}^{\alpha }\right]\cdot 1_{\left[p,1\right]}\left(v\right),\;\alpha >0,\;\alpha \neq 1,\\ \varpi_{p,\lambda }\left(v\right) & =\left\{\frac{1}{1-p}-\frac{\lambda }{\alpha -1}\cdot \left[\frac{-1}{1-p}+\frac{\alpha }{1-p}{\left(\frac{1-v}{1-p}\right)}^{\alpha -1}\right]\right\}\cdot 1_{\left[p,1\right]}\left(v\right),\;\alpha >0,\;\alpha \neq 1. \end{array}$$
*(2)*  *${\mathrm{CRTES}}_{\alpha ,p}^{\lambda }$ satisfies translation invariance and positive homogeneous and comonotonic additive properties.*
*(3)*  *The below statements are equivalent: (i) ${\mathrm{CRTES}}_{\alpha ,p}^{\lambda }$ satisfies the monotone property; (ii) ${\mathrm{CRTES}}_{\alpha ,p}^{\lambda }$ satisfies the sub-additive property; (iii) ${\mathrm{CRTES}}_{\alpha ,p}^{\lambda }$ holds the increasing convex order; (iv) ${\mathrm{CRTES}}_{\alpha ,p}^{\lambda }$ is a coherent risk measure; (v) λ∈[0,1].*

**Proof.** 

(1) Using (1) and (23) of ${\mathrm{ES}}_{p}\left(X\right)$ and ${\mathrm{TCRTE}}_{\alpha ,p}\left(X\right)$, we immediately obtain (27) and (28).
(2) From (23), the positive homogeneous and comonotonic additive properties of ${\mathrm{CRTES}}_{\alpha ,p}^{\lambda }$ are obtained. Further, since
  $${\mathrm{ES}}_{p}\left(X+c\right)={\mathrm{ES}}_{p}\left(X\right)+c,{\mathrm{TCRTE}}_{\alpha ,p}\left(X+c\right)={\mathrm{TCRTE}}_{\alpha ,p}\left(X\right),\forall c\in R,$$
  the translation invariance of ${\mathrm{CRTES}}_{\alpha ,p}^{\lambda }$ follows.
(3) Noting that $\varpi_{p,\lambda }\left(v\right)=0$ for all v∈[0,p), and that $\varpi_{p,\lambda }\left(v\right)$ is an increasing function on [p,1], therefore, we have that $\varpi_{p,\lambda }\left(v\right)$ is non-negative if and only if λ∈[0,1]. Furthermore, $\varpi_{p,\lambda }\left(v\right)$ is non-decreasing if and only if λ∈[0,1]. Using Lemma 4.2 of [5], one obtains that ${\mathrm{CRTES}}_{\alpha ,p}^{\lambda }$ satisfies the monotone property if and only if $\varpi_{p,\lambda }\left(v\right)$ for all v∈[0,1], and that ${\mathrm{CRTES}}_{\alpha ,p}^{\lambda }$ is sub-additive if and only if $\varpi_{p,\lambda }\left(v\right)$ is increasing in v∈[0,1]. Therefore, (i)⇔(ii)⇔(v).

Next, by Theorem 2.1 of [38] and (28), we know that if $\varpi_{p,\lambda }\left(v\right)$ is increasing in v∈[0,1], then (iii) follows; that is to say, (ii)⇒(iii)⇒(i). Then, (iii)⇔(i).

Furthermore, by Corollary 4.65 of [3], we learn that a law-invariant coherent risk measure holds the increasing convex order. This reveals that (iv)⇒(iii), then (iv)⇒(v). On the contrary, since ${\mathrm{CRTES}}_{\alpha ,p}^{\lambda }$ satisfies translation invariance and positive homogeneous properties, and (v)⇔(i)⇔(ii), we have (v)⇒(iv). Hence, (iv)⇔(v). □

**Remark** **5.** *Let α=2 in Theorem 5; we obtain tail-Gini shortfall (see [6,39]), which is different from the results in [5]. In addition, let α→1 in Theorem 5; we obtain the CRE shortfall in [6].*

**Theorem** **6.** *Assume that p∈(0,1), λ∈[0,∞) and the convex cone $X=L^{2}$.*

*(1)*  *${\mathrm{RTDS}}_{p}^{\lambda }$ is represented as:*
$$\begin{array}{cc} {\mathrm{RTDS}}_{p}^{\lambda }\left(X\right) & =\int_{0}^{1}F_{X}^{-1}\left(v\right)dg_{p,\lambda }\left(v\right)=\int_{0}^{1}F_{X}^{-1}\left(v\right)\varpi_{p,\lambda }\left(v\right)dv, \end{array}$$
  *where*
$$\begin{array}{cc} g_{p,\lambda }\left(v\right) & =\frac{1}{1-p}{\left(v-p\right)}_{+}-\lambda \cdot \left[{\left(\frac{1-v}{1-p}\right)}^{1/2}-\left(\frac{1-v}{1-p}\right)\right]\cdot 1_{\left[p,1\right]}\left(v\right),\\ \varpi_{p,\lambda }\left(v\right) & =\left\{\frac{1}{1-p}+\frac{\lambda }{1-p}\cdot \left[\frac{1}{2}{\left(\frac{1-v}{1-p}\right)}^{-1/2}-1\right]\right\}\cdot 1_{\left[p,1\right]}\left(v\right). \end{array}$$
*(2)*  *${\mathrm{RTDS}}_{p}^{\lambda }$ satisfies translation invariance and positive homogeneous and comonotonic additive properties.*
*(3)*  *The below statements are equivalent: (i) ${\mathrm{RTDS}}_{p}^{\lambda }$ satisfies the monotone property; (ii) ${\mathrm{RTDS}}_{p}^{\lambda }$ satisfies the sub-additive property; (iii) ${\mathrm{RTDS}}_{p}^{\lambda }$ holds the increasing convex order; (iv) ${\mathrm{RTDS}}_{p}^{\lambda }$ is a coherent risk measure; (v) λ∈[0,2].*

**Proof.**  Let $\alpha =\frac{1}{2}$ in Theorem 5; combining with (25), we obtain the desired results. □

**Theorem** **7.** *Assume that U∼U(0,1) and $X^{\alpha }\in L^{2}$ are independent. Then,*

(29) $$\begin{array}{c} {\mathrm{TCRTE}}_{\alpha ,p}\left(X\right)=\alpha E\left[{\left(\frac{1-U}{1-p}\right)}^{\alpha -1}{\mathrm{ES}}_{U}\left(X\right)|U>p\right]-{\mathrm{ES}}_{p}\left(X\right). \end{array}$$

*Furthermore,*

(30) $$\begin{array}{c} {\mathrm{CRTES}}_{\alpha ,p}^{\lambda }\left(X\right)=\lambda \alpha E\left[{\left(\frac{1-U}{1-p}\right)}^{\alpha -1}{\mathrm{ES}}_{U}\left(X\right)|U>p\right]+\left(1-\lambda \right){\mathrm{ES}}_{p}\left(X\right). \end{array}$$

**Proof.**  By (23), we have

(31) $$\begin{array}{cc} {\mathrm{TCRTE}}_{\alpha ,p}\left(X\right)\; & =\frac{-\alpha }{\alpha -1}\mathrm{Cov}\left[F_{X}^{-1}\left(U\right),{\left(\frac{1-U}{1-p}\right)}^{\alpha -1}|U>p\right]\\ & =\frac{-\alpha }{\alpha -1}\left\{\frac{1}{1-p}\int_{p}^{1}F_{X}^{-1}\left(u\right){\left(\frac{1-u}{1-p}\right)}^{\alpha -1}du-{\mathrm{ES}}_{p}\left(X\right)\cdot E\left[{\left(\frac{1-U}{1-p}\right)}^{\alpha -1}|U>p\right]\right\}\\ \; & =\frac{-\alpha }{\left(\alpha -1)(1-p\right)}\int_{p}^{1}F_{X}^{-1}\left(u\right){\left(\frac{1-u}{1-p}\right)}^{\alpha -1}du+\frac{1}{\alpha -1}{\mathrm{ES}}_{p}\left(X\right), \end{array}$$

where the last equality follows by using relation

$$\begin{array}{c} E\left[{\left(\frac{1-U}{1-p}\right)}^{\alpha -1}|U>p\right]=\frac{1}{\alpha }. \end{array}$$

Note that

$$\begin{array}{cc} \frac{-\alpha }{\left(\alpha -1)(1-p\right)}\int_{p}^{1}F_{X}^{-1}\left(u\right){\left(\frac{1-u}{1-p}\right)}^{\alpha -1}du\; & =\frac{-\alpha }{\left(\alpha -1\right){\left(1-p\right)}^{\alpha }}\int_{p}^{1}F_{X}^{-1}\left(u\right)\int_{u}^{1}\left(\alpha -1\right){\left(1-v\right)}^{\alpha -2}dvdu\\ \; & =\frac{-\alpha }{{\left(1-p\right)}^{\alpha }}\int_{p}^{1}\int_{p}^{v}F_{X}^{-1}\left(u\right){\left(1-v\right)}^{\alpha -2}dudv\\ \; & =\frac{-\alpha }{{\left(1-p\right)}^{\alpha }}\int_{p}^{1}\left[\left(1-p\right){\mathrm{ES}}_{p}\left(X\right)-\left(1-v\right){\mathrm{ES}}_{v}\left(X\right)\right]{\left(1-v\right)}^{\alpha -2}dv\\ \; & =\frac{-\alpha }{\alpha -1}{\mathrm{ES}}_{p}\left(X\right)+\alpha E\left[{\left(\frac{1-U}{1-p}\right)}^{\alpha -1}{\mathrm{ES}}_{U}\left(X\right)|U>p\right], \end{array}$$

combining with (31), we obtain (29). Therefore, we obtain (30), ending the proof. □

**Theorem** **8.** *Assume that U∼U(0,1) and $X\in L^{2}$ are independent. Then,*

(32) $$\begin{array}{c} {\mathrm{TRTD}}_{p}\left(X\right)=\frac{1}{4}E\left[{\left(\frac{1-U}{1-p}\right)}^{-1/2}{\mathrm{ES}}_{U}\left(X\right)|U>p\right]-{\mathrm{ES}}_{p}\left(X\right). \end{array}$$

*Furthermore,*

(33) $$\begin{array}{c} {\mathrm{RTDS}}_{p}^{\lambda }\left(X\right)=\frac{\lambda }{4}E\left[{\left(\frac{1-U}{1-p}\right)}^{-1/2}{\mathrm{ES}}_{U}\left(X\right)|U>p\right]+\left(1-\lambda \right){\mathrm{ES}}_{p}\left(X\right). \end{array}$$

**Proof.**  Let $\alpha =\frac{1}{2}$ in Theorem 7; combining with (25), we obtain the desired results. □

## 5. ${\mathrm{CRTES}}_{\alpha ,p}^{\lambda }$ for Some Distributions

### 5.1. Elliptical Distributions

Consider an elliptical random variable $X∼E_{1}\left(\mu ,\sigma^{2},g_{1}\right)$. If the probability density function (pdf) of *X* exists, its form will be (see [40])

$$\begin{array}{c} f_{X}\left(v\right):=\frac{c_{1}}{\sigma }g_{1}\left\{\frac{1}{2}{\left(\frac{v-\mu }{\sigma }\right)}^{2}\right\},\;v\in R, \end{array}$$

where μ and σ>0 are location and scale parameters, respectively. Moreover, $g_{1}\left(t\right)$, t≥0, is the density generator of *X*, and is denoted by $X∼E_{1}\left(\mu ,\;\sigma^{2},\;g_{1}\right)$. The density generator $g_{1}$ satisfies the condition

$$\begin{array}{c} \int_{0}^{\infty }s^{-1/2}g_{1}\left(s\right)ds<\infty , \end{array}$$

and the normalizing constant $c_{1}$ is given by

$$\begin{array}{cc} c_{1} & =\frac{1}{\sqrt{2}}{\left[\int_{0}^{\infty }s^{-1/2}g_{1}\left(s\right)ds\right]}^{-1}. \end{array}$$

Cumulative generator ${\bar{G}}_{1}\left(u\right)$ and normalizing constant $c_{1}^{*}$ are, respectively, defined as follows:

$$\begin{array}{c} {\bar{G}}_{1}\left(u\right)=\int_{u}^{\infty }g_{1}\left(v\right)dv \end{array}$$

and

$$\begin{array}{c} c_{1}^{*}=\frac{1}{\sqrt{2}}{\left[\int_{0}^{\infty }s^{-1/2}{\bar{G}}_{1}\left(s\right)ds\right]}^{-1}<\infty . \end{array}$$

Landsman and Valdez [40] proved that

$$\begin{array}{c} {\mathrm{ES}}_{p}\left(X\right)=\mu +\frac{c_{1}\sigma {\bar{G}}_{1}\left(\frac{1}{2}w_{p}^{2}\right)}{1-p}, \end{array}$$

where $w_{p}=\frac{x_{p}-\mu }{\sigma }$.

Now, several important cases, including normal, Student-*t*, logistic and Laplace distributions, are given as follows.

**Example** **21.** *(Normal distribution) Let $X∼N_{1}\left(\mu ,\;\sigma^{2}\right)$. In this case, the density generators are written as*

$$\begin{array}{c} g_{1}\left(s\right)={\bar{G}}_{1}\left(s\right)=\exp\left\{-s\right\}, \end{array}$$

*and the normalization constants are given by*

$$\begin{array}{c} c_{1}=c_{1}^{*}={\left(2\pi \right)}^{-\frac{1}{2}}. \end{array}$$

*Then,*

(34) $$\begin{array}{c} {\mathrm{ES}}_{p}\left(X\right)=\mu +\frac{{\left(2\pi \right)}^{-\frac{1}{2}}\sigma \exp\left(-\frac{1}{2}w_{p}^{2}\right)}{1-p}, \end{array}$$

*where $w_{p}=\frac{x_{p}-\mu }{\sigma }$.*

Without loss of generality [for the convenience of simulation], let $X∼N_{1}\left(0,\;1\right)$; by Equations (29), (30) and (34), we can use the R software to compute ${\mathrm{TCRTE}}_{\alpha ,p}\left(X\right)$ and ${\mathrm{CRTES}}_{\alpha ,p}^{\lambda }\left(X\right)$ for p∈[0.9,1), and the results are shown in Figure 2.

From Figure 2a, we find that when α is fixed, ${\mathrm{TCRTE}}_{\alpha ,p}$ is decreasing in *p*. Moreover, when *p* is fixed, ${\mathrm{TCRTE}}_{\alpha ,p}$ is also decreasing in α. As we can see in Figure 2b, when α is fixed, ${\mathrm{CRTES}}_{\alpha ,p}^{\lambda }$ is increasing in *p*, while ${\mathrm{CRTES}}_{\alpha ,p}^{\lambda }$ will be decreasing in α when *p* is fixed. In Figure 2c, we observe that when λ is fixed, ${\mathrm{CRTES}}_{\alpha ,p}^{\lambda }$ is increasing in *p*. Moreover, when *p* is fixed, ${\mathrm{TCRTE}}_{\alpha ,p}$ is also increasing in λ.

**Example** **22.** *(Student-t distribution). Let $X∼St_{1}\left(\mu ,\;\sigma^{2},\;m\right)$. In this case, the density generators are written as*

$$\begin{array}{c} g_{1}\left(s\right)={\left(1+\frac{2s}{m}\right)}^{-(m+1)/2},\;\mathrm{and}\;{\bar{G}}_{1}\left(s\right)=\frac{m}{m-1}{\left(1+\frac{2s}{m}\right)}^{-(m-1)/2}. \end{array}$$

*The normalization constants are given by*

$$\begin{array}{cc} c_{1}=\frac{\Gamma \left((m+1)/2\right)}{\Gamma \left(m/2\right){\left(m\pi \right)}^{\frac{1}{2}}},\;\mathrm{and}\;c_{1}^{*} & =\frac{\left(m-1\right)}{\sqrt{2}m}{\left[\int_{0}^{\infty }s^{1/2-1}{\left(1+\frac{2t}{m}\right)}^{-(m-1)/2}ds\right]}^{-1}=\frac{\left(m-1\right)}{m^{3/2}B\left(\frac{1}{2},\;\frac{m-2}{2}\right)},\;\mathrm{if}\;m>2, \end{array}$$

*where Γ(·) and B(·,·) denote gamma and beta functions, respectively. Then,*

(35) $$\begin{array}{c} {\mathrm{ES}}_{p}\left(X\right)=\mu +\frac{\sigma m}{\left(1-p)(m-1\right)}\frac{\Gamma \left((m+1)/2\right)}{\Gamma \left(m/2\right){\left(m\pi \right)}^{\frac{1}{2}}}{\left(1+\frac{w_{p}^{2}}{m}\right)}^{-(m-1)/2}, \end{array}$$

*where $w_{p}=\frac{x_{p}-\mu }{\sigma }$.*

Let $X∼St_{1}\left(0,\;1,\;m\right)$; by Equations (29), (30) and (35), we can use the R software to compute ${\mathrm{TCRTE}}_{\alpha ,p}\left(X\right)$ and ${\mathrm{CRTES}}_{\alpha ,p}^{\lambda }\left(X\right)$ for p∈[0.9,1), and the results are shown in Figure 3.

From Figure 3a, we find that the degree of freedom, m, has a great impact on ${\mathrm{TCRTE}}_{\alpha ,p}$. ${\mathrm{TCRTE}}_{\alpha ,p}$ is decreasing in *m*. When *m* is small, ${\mathrm{TCRTE}}_{\alpha ,p}$ is increasing in *p*, while ${\mathrm{TCRTE}}_{\alpha ,p}$ will be decreasing in *p* instead of increasing when *m* is larger than a threshold. From Figure 3b, we find that when α is fixed, ${\mathrm{TCRTE}}_{\alpha ,p}$ is increasing in *p*. However, when *p* is fixed, ${\mathrm{TCRTE}}_{\alpha ,p}$ is decreasing in α. In Figure 3c, we observe that when α is fixed, ${\mathrm{CRTES}}_{\alpha ,p}^{\lambda }$ is increasing in *p*. However, when *p* is fixed, ${\mathrm{CRTES}}_{\alpha ,p}^{\lambda }$ is decreasing in α. From Figure 3d, we find that when λ is fixed, ${\mathrm{CRTES}}_{\alpha ,p}^{\lambda }$ is increasing in *p*. Moreover, when *p* is fixed, ${\mathrm{CRTES}}_{\alpha ,p}^{\lambda }$ is also increasing in λ.

**Example** **23.** *(Logistic distribution). Let $X∼Lo_{1}\left(\mu ,\;\sigma^{2}\right)$. In this case, the density generators are written as*

$$\begin{array}{c} g_{1}\left(s\right)=\frac{\exp(-\sqrt{2s})}{{\left[1+\exp(-\sqrt{2s})\right]}^{2}},\;\mathrm{and}\;{\bar{G}}_{1}\left(s\right)=\frac{\sqrt{2s}\exp\left(-\sqrt{2s}\right)}{1+\exp(-\sqrt{2s})}+\log\left(1+\exp(-\sqrt{2s})\right). \end{array}$$

*The normalization constants are given by*

$$\begin{array}{c} c_{1}=1,\;\mathrm{and}\;c_{1}^{*}=\frac{1}{\sqrt{2}}{\left\{\int_{0}^{\infty }s^{-1/2}\left[\frac{\sqrt{2s}\exp\left(-\sqrt{2s}\right)}{1+\exp(-\sqrt{2s})}+\log\left(1+\exp(-\sqrt{2s})\right)\right]ds\right\}}^{-1}. \end{array}$$

*Then,*

(36) $$\begin{array}{cc} {\mathrm{ES}}_{p}\left(X\right) & =\mu +\frac{\sigma }{1-p}\left[\frac{w_{p}\exp\left(-w_{p}\right)}{1+\exp(-w_{p})}+\log\left(1+\exp(-w_{p})\right)\right], \end{array}$$

*where $w_{p}=\frac{x_{p}-\mu }{\sigma }$.*

Let $X∼Lo_{1}\left(0,\;1\right)$; by Equations (29), (30) and (36), we can use the R software to compute ${\mathrm{TCRTE}}_{\alpha ,p}\left(X\right)$ and ${\mathrm{CRTES}}_{\alpha ,p}^{\lambda }\left(X\right)$ for p∈[0.9,1), and the results are shown in Figure 4.

It is seen from Figure 4a that the α has a little impact on the values of ${\mathrm{TCRTE}}_{\alpha ,p}$. For fixed *p*, ${\mathrm{TCRTE}}_{\alpha ,p}$ is decreasing in α. From Figure 4b, we observe that when α is fixed, ${\mathrm{CRTES}}_{\alpha ,p}^{\lambda }$ is increasing in *p*. However, when *p* is fixed, ${\mathrm{CRTES}}_{\alpha ,p}^{\lambda }$ is decreasing in α. In Figure 4c, we find that when λ is fixed, ${\mathrm{CRTES}}_{\alpha ,p}^{\lambda }$ is increasing in *p*. Moreover, when *p* is fixed, ${\mathrm{CRTES}}_{\alpha ,p}^{\lambda }$ is also increasing in λ.

**Example** **24.** *(Laplace distribution). Let $X∼La_{1}\left(\mu ,\;\sigma^{2}\right)$. In this case, the density generators are written as*

$$\begin{array}{c} g_{1}\left(s\right)=\exp\left(-\sqrt{2s}\right), \end{array}$$

*and*

$$\begin{array}{c} {\bar{G}}_{1}\left(s\right)=\left(1+\sqrt{2s}\right)\exp\left(-\sqrt{2s}\right). \end{array}$$

*The corresponding normalization constants are given by*

$$\begin{array}{c} c_{1}=\frac{1}{2},\;\mathrm{and}\;c_{1}^{*}=\frac{1}{4}. \end{array}$$

*Then,*

(37) $$\begin{array}{c} {\mathrm{ES}}_{p}\left(X\right)=\mu +\frac{\sigma (1+|w_{p}\left|)\exp(-\right|w_{p}\left|\right)}{2(1-p)}, \end{array}$$

*where $w_{p}=\frac{x_{p}-\mu }{\sigma }$.*

Let $X∼La_{1}\left(0,\;1\right)$; by Equations (29), (30) and (37), we can use the R software to compute ${\mathrm{TCRTE}}_{\alpha ,p}\left(X\right)$ and ${\mathrm{CRTES}}_{\alpha ,p}^{\lambda }\left(X\right)$ for p∈[0.9,1), and the results are shown in Figure 5.

It is seen from Figure 5a that *p* has almost no impact on ${\mathrm{TCRTE}}_{\alpha ,p}$. For fixed α, ${\mathrm{TCRTE}}_{\alpha ,p}$ is almost the same in *p*. However, α has a great impact on ${\mathrm{TCRTE}}_{\alpha ,p}$. For fixed *p*, ${\mathrm{TCRTE}}_{\alpha ,p}$ is decreasing in α. In Figure 5b, we observe that when α is fixed, ${\mathrm{CRTES}}_{\alpha ,p}^{\lambda }$ is increasing in *p*. However, when *p* is fixed, ${\mathrm{CRTES}}_{\alpha ,p}^{\lambda }$ is decreasing in α. From Figure 5c, we find that when λ is fixed, ${\mathrm{CRTES}}_{\alpha ,p}^{\lambda }$ is increasing in *p*. Moreover, when *p* is fixed, ${\mathrm{CRTES}}_{\alpha ,p}^{\lambda }$ is also increasing in λ.

### 5.2. Inverse Gaussian, Gamma and Beta Distributions

**Example** **25.** *(Inverse Gaussian distribution) An inverse Gaussian random variable X∼IG(β,μ), with parameters β>0 and μ>0, has its probability density function (pdf) as*

$$f\left(x;\beta ,\mu \right)=\sqrt{\frac{\beta }{2\pi x^{3}}}\exp\left\{-\frac{\beta {\left(x-\mu \right)}^{2}}{2\mu^{2}x}\right\},\;x>0.$$

*From Example 4.3 of Landsman and Valdez (2005), we can obtain*

(38) $$\begin{array}{c} {\mathrm{ES}}_{p}\left(X\right)=\mu +\frac{\mu }{\beta (1-p)}\left\{\sqrt{\beta x_{p}}\phi \left(a_{p}\right)+\exp\left(\frac{2\beta }{\mu }\right)\left[2\beta \Phi \left(b_{p}\right)-\sqrt{\beta x_{p}}\phi \left(b_{p}\right)\right]\right\}, \end{array}$$

*where $a_{p}=\frac{\sqrt{\beta x_{p}}}{\mu }-\sqrt{\frac{\beta }{x_{p}}}$, $b_{p}=-\frac{\sqrt{\beta x_{p}}}{\mu }-\sqrt{\frac{\beta }{x_{p}}}$, and $x_{p}$ is the pth quantile of X.*

Let $X∼IG\left(10,\;1\right)$; by Equations (29), (30) and (38), we can use the R software to compute ${\mathrm{TCRTE}}_{\alpha ,p}\left(X\right)$ and ${\mathrm{CRTES}}_{\alpha ,p}^{\lambda }\left(X\right)$ for p∈[0.9,1), and the results are shown in Figure 6.

**Example** **26.** *(Gamma distribution) A random variable X∼Γ(β,γ), with parameters β>0 and γ>0, follows Gamma distribution if its pdf is*

$$f\left(x;\beta ,\gamma \right)=\frac{x^{\beta -1}}{\Gamma (\beta )}\exp\left\{-\gamma x+\beta \log\gamma \right\},\;x>0.$$

*Landsman and Valdez [41] provided that*

(39) $$\begin{array}{c} {\mathrm{ES}}_{p}\left(X\right)=\frac{\beta \bar{F}\left(x_{p};\beta +1,\gamma \right)}{\gamma (1-p)}, \end{array}$$

*where $\bar{F}\left(x_{p};\beta +1,\gamma \right)=1-F\left(x_{p};\beta +1,\gamma \right)$ is the tail distribution function of Y∼Γ(β+1,γ), and $x_{p}$ is the pth quantile of X.*

Let $X∼\Gamma \left(5,\;1\right)$; by Equations (29), (30) and (39), we can use the R software to compute ${\mathrm{TCRTE}}_{\alpha ,p}\left(X\right)$ and ${\mathrm{CRTES}}_{\alpha ,p}^{\lambda }\left(X\right)$ for p∈[0.9,1), and the results are shown in Figure 7.

**Example** **27.** *(Beta distribution) A random variable X∼B(β,γ), with parameters β>0 and γ>0, follows Beta distribution if its pdf is*

$$f\left(x;\beta ,\gamma \right)=\frac{\Gamma (\beta +\gamma )}{\left(\Gamma (\beta )\Gamma (\gamma )\right)}x^{\beta -1}{\left(1-x\right)}^{\gamma -1},\;0<x<1.$$

*We can obtain*

(40) $$\begin{array}{c} {\mathrm{ES}}_{p}\left(X\right)=\frac{\beta \bar{F}\left(x_{p};\beta +1,\gamma \right)}{\left(\beta +\gamma )(1-p\right)}, \end{array}$$

*where $\bar{F}\left(x_{p};\beta +1,\gamma \right)=1-F\left(x_{p};\beta +1,\gamma \right)$ is the tail distribution function of Y∼B(β+1,γ), and $x_{p}$ is the pth quantile of X.*

Let $X∼B\left(5,\;2\right)$; by Equations (29), (30) and (40), we can use the R software to compute ${\mathrm{TCRTE}}_{\alpha ,p}\left(X\right)$ and ${\mathrm{CRTES}}_{\alpha ,p}^{\lambda }\left(X\right)$ for p∈[0.9,1), and the results are shown in Figure 8.

## 6. Concluding Remarks

This paper has derived covariance and Choquet integral representations of some entropies, and has proposed shortfalls of entropy CRTES and RTDS. In particular, CRTESs of elliptical, inverse Gaussian, gamma and beta distributions are computed. Furthermore, Hou and Wang [42] generalized the tail-Gini functional of a random variable to a case of a two-dimensional random vector, and Sun et al. [8] extended the TCRE to the two risks (random vector). In the future, we will try to extend the TCRTE and TRTD of a random variable in this paper to a two-dimensional random vector.

## Data Availability

Not applicable.
